# Exploring the Potential of Multinuclear Solid‐State ^1^H, ^13^C, and ^35^Cl Magnetic Resonance To Characterize Static and Dynamic Disorder in Pharmaceutical Hydrochlorides

**DOI:** 10.1002/cphc.202200558

**Published:** 2022-11-07

**Authors:** Patrick M. J. Szell, Zainab Rehman, Ben P. Tatman, Leslie P. Hughes, Helen Blade, Steven P. Brown

**Affiliations:** ^1^ Department of Physics University of Warwick Coventry CV4 7AL UK; ^2^ Oral Product Development Pharmaceutical Technology & Development, Operations AstraZeneca Macclesfield SK10 2NA UK

**Keywords:** crystallographic disorder, density functional theory, NMR, pharmaceuticals, X-ray crystallography

## Abstract

Crystallographic disorder, whether static or dynamic, can be detrimental to the physical and chemical stability, ease of crystallization and dissolution rate of an active pharmaceutical ingredient. Disorder can result in a loss of manufacturing control leading to batch‐to‐batch variability and can lengthen the process of structural characterization. The range of NMR active nuclei makes solid‐state NMR a unique technique for gaining nucleus‐specific information about crystallographic disorder. Here, we explore the use of high‐field ^35^Cl solid‐state NMR at 23.5 T to characterize both static and dynamic crystallographic disorder: specifically, dynamic disorder occurring in duloxetine hydrochloride (**1**), static disorder in promethazine hydrochloride (**2**), and trifluoperazine dihydrochloride (**3**). In all structures, the presence of crystallographic disorder was confirmed by ^13^C cross‐polarization magic‐angle spinning (CPMAS) NMR and supported by GIPAW‐DFT calculations, and in the case of **3**, ^1^H solid‐state NMR provided additional confirmation. Applying ^35^Cl solid‐state NMR to these compounds, we show that higher magnetic fields are beneficial for resolving the crystallographic disorder in **1** and **3**, while broad spectral features were observed in **2** even at higher fields. Combining the data obtained from ^1^H, ^13^C, and ^35^Cl NMR, we show that **3** exhibits a unique case of disorder involving the ^+^N−H hydrogen positions of the piperazinium ring, driving the chloride anions to occupy three distinct sites.

## Graphical abstract

^35^Cl solid‐state NMR is used to investigate the crystallographic disorder occurring in pharmaceutical hydrochloride salts, uncovering a unique case of proton‐driven disorder of the chloride anion in trifluoperazine dihydrochloride.

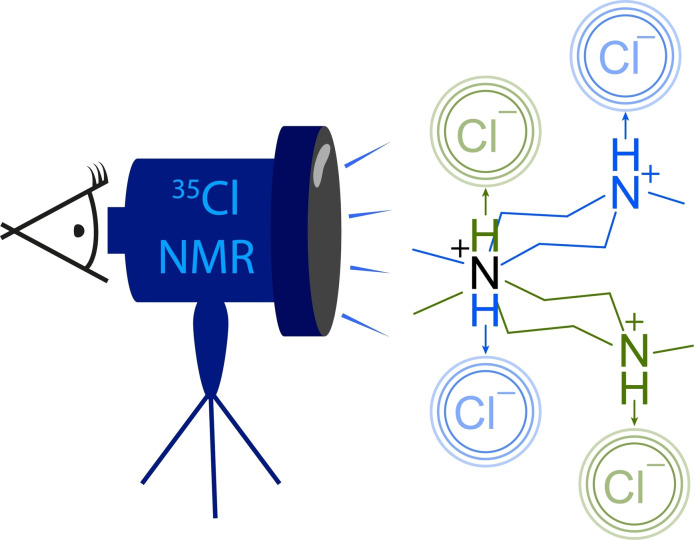

## Introduction

Crystallographic disorder has been shown to render the crystallization process more difficult, and plays a role in the properties of an active pharmaceutical ingredient (API).^[1]^ In addition, crystallographic disorder occurring in the solid form of pharmaceuticals also introduces risks during the process of structural characterization and manufacturing.^[2]^ For instance, crystallographic disorder can arise in part due to conformational degrees of freedom in the molecule, e. g. static disorder, and/or due to the occurrence of molecular motion, e. g. dynamic disorder.^[3]^ While there are simple cases of dynamic disorder, such as the methyl group rotation, there have also been reports of larger moieties exhibiting dynamic disorder, thereby introducing complications during the process of structural modelling.^[4]^

Crystallographic disorder raises several major challenges in terms of structural characterization, with X‐ray crystallography typically being the current tool of choice. Unfortunately, crystallographic disorder can make crystal growth more difficult and complicate the interpretation of the X‐ray data. Further, low‐temperature X‐ray data may not observe dynamics taking place at room temperature. Solid‐state NMR spectroscopy is routinely applied to powdered samples and offers useful crystallographic information on disordered molecules *via* chemical shifts, quadrupolar coupling, or even dipolar coupling,[^3a^, ^5^] under the theme of NMR crystallography.^[6]^ Solid‐state NMR offers nucleus‐specific information from several pharmaceutically‐relevant nuclei (e. g. ^1^H, ^13^C, ^14/15^N, ^17^O, ^23^Na, ^35^Cl), and variable‐temperature NMR experiments have been shown to be powerful for characterizing the occurrence of dynamics that may otherwise have gone unnoticed.^[5a]^ A recent review by Li et al. details the advantages of solid‐state NMR for the analysis of pharmaceuticals.^[7]^

With several APIs formulated in their hydrochloride forms,^[8]^ solid‐state NMR observing the ^35^Cl nucleus (spin *I*=3/2, *Q*=−81.12 mb)^[9]^ has been shown to be a versatile opportunity to characterize these solid forms.[^6l^, ^10^] As a quadrupolar nucleus, ^35^Cl solid‐state NMR can provide valuable information on the chemical shielding tensor^[11]^ as well as the quadrupolar coupling tensor,^[12]^ which can potentially be exploited to characterize crystallographic disorder. The information obtained from ^35^Cl solid‐state NMR has been shown to be a powerful approach to characterizing pharmaceuticals and its hydrates.[^6l^, ^10^] Further, ^35^Cl NMR has been used for tracking disproportionation in formulated tablets,^[10g]^ investigating non‐covalent interactions,^[13]^ and for gaining information on the crystallography.[^10b^, ^10d^, ^10f^, ^14^]

**Figure 1:**
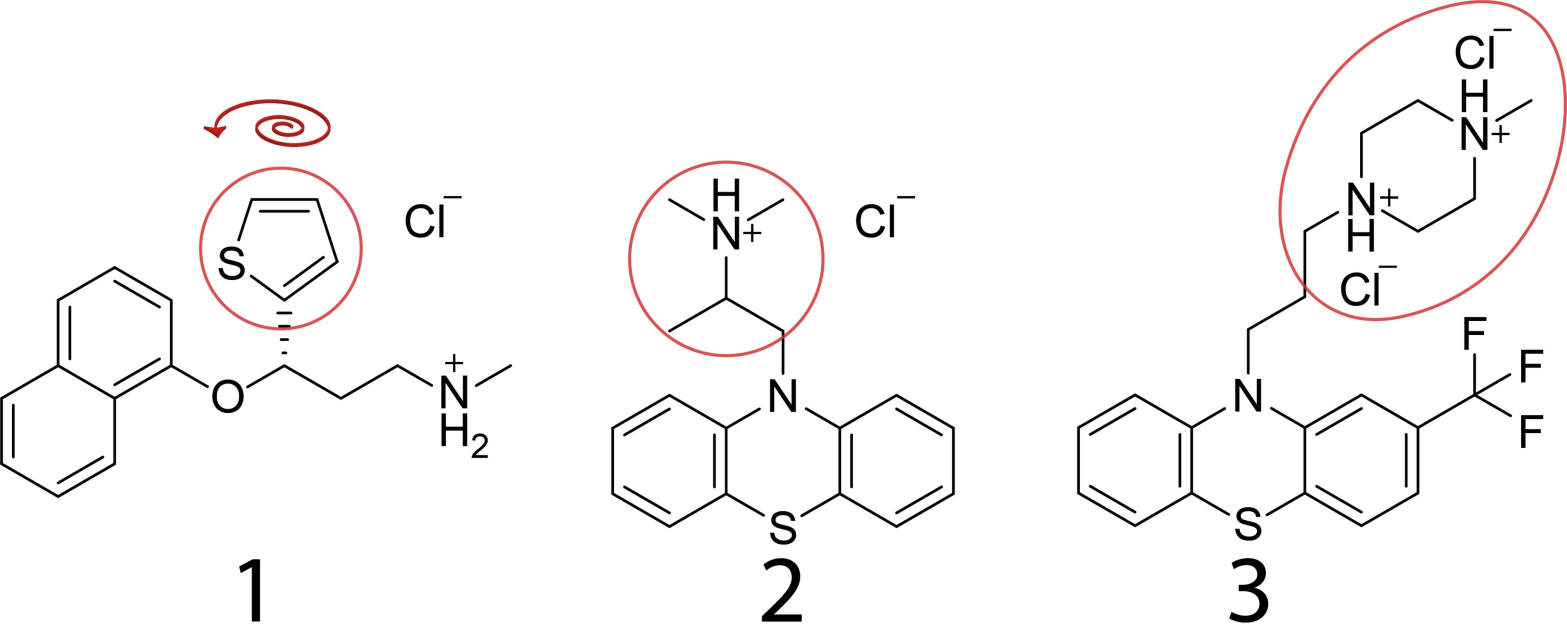
The molecular structure of duloxetine hydrochloride (**1**), promethazine hydrochloride (**2**), and trifluoperazine dihydrochloride (**3**). The red circle denotes the moieties exhibiting crystallographic disorder, and the red arrow above **1** denotes the rotation of the thiophene group.

While crystallographic disorder often results in the broadening of the ^35^Cl NMR lineshape, there has been some previous work where ^35^Cl NMR was applied to investigate disordered structures. These include, for instance, the long‐range disorder in polymorphs of Mexiletine hydrochloride,[^10d^, ^10f^] a pharmaceutical compound under development,^[10a]^ disorder in the Ziegler‐Natta catalyst,^[15]^ and chloride anions exhibiting dynamics in water environments.^[16]^ Here, we investigate three hydrochloride salts exhibiting various types of crystallographic disorder: duloxetine hydrochloride (**1**), promethazine hydrochloride (**2**), and trifluoperazine dihydrochloride (**3**) (see molecular structures in Figure 1). The crystallographic disorder is nearby to the chloride anion and arises due to dynamic disorder (**1**), and static disorder (**2**, **3**). In addition to nearby static disorder, the chloride anion is also disordered in **3**. In this work, each sample is investigated by ^1^H, ^1^H−^13^C CPMAS,^[17]^ and ^35^Cl solid‐state NMR, and is supported by gauge‐included projector augmented wave (GIPAW)^[18]^ density functional theory (DFT) calculations. While ^1^H and ^13^C solid‐state NMR provide the first‐line characterization of the disorder in **1**–**3**, the proximity of the chloride anions also enables us to use ^35^Cl solid‐state NMR as a probe.

## Results and Discussion

### X‐ray Crystallography and Optimizations

**Figure 2:**
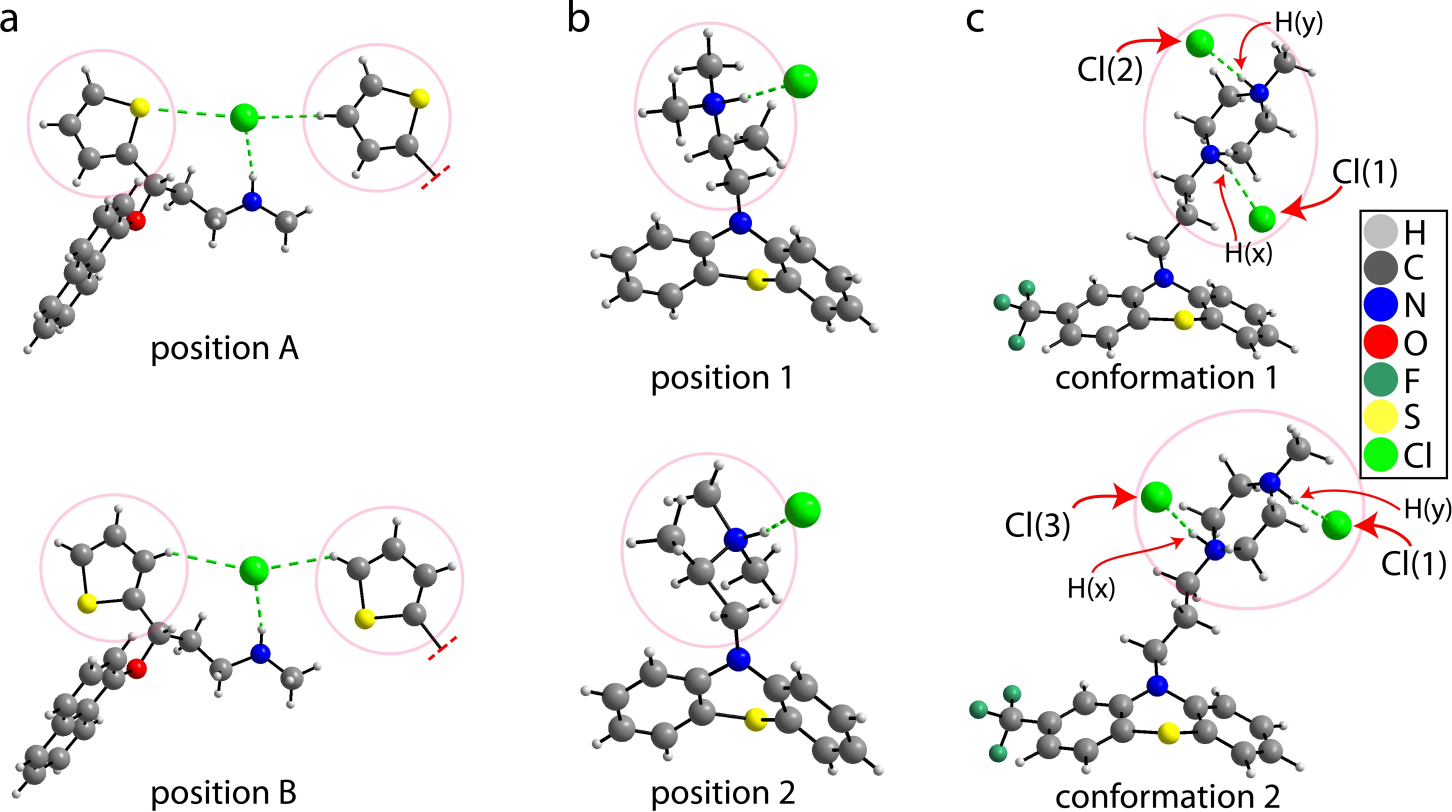
Depiction of the X‐ray crystal structure of a) **1**, b) **2**, and c) **3**. The two disordered positions for each structure were separated above and below, and the pink circles highlight moieties exhibiting crystallographic disorder. The three unique chloride positions in **3** are labelled Cl(1), Cl(2), and Cl(3), and the two hydrogen atoms added during the structural modelling have been labelled H(x) and H(y).

All X‐ray structures used in this study are summarized in Table S1 of the Supporting Information. The X‐ray crystal structure of **1** was previously reported and discussed by Bhadbhade et al., and a full description of the structure can be found in their original report.^[19]^ The structure features a single molecule and chloride anion in the asymmetric unit (*Z*′=1), with the thiophene group of **1** disordered over two positions in an occupancy ratio of 0.58 : 0.42. In a previously reported variable‐temperature ^13^C solid‐state NMR investigation, the disorder was found to be dynamic in nature.^[20]^ As shown in Figure S1 of the Supporting Information, the coordination sphere surrounding the chloride anion includes a ^+^N−H⋅⋅⋅Cl^−^ hydrogen bond (*d*_H1A⋅⋅⋅Cl_=2.23 Å, *d*_N⋅⋅⋅Cl_=3.11 Å, θ_N‐H1A⋅⋅⋅Cl_=161°; *d*_H1B⋅⋅⋅Cl_=2.18 Å, *d*_N⋅⋅⋅Cl_=3.09 Å, θ_N‐H1B⋅⋅⋅Cl_=170°) and several close H⋅⋅⋅Cl^−^ and S⋅⋅⋅Cl^−^ contacts.^[19]^ Interestingly, the chloride anion is positioned between two disordered thiophene rings, with the sulphur atom either pointing towards or away from the chloride anion, as shown in Figure 2a.

The X‐ray crystal structure of **2** has been previously reported by Borodi et al., finding a rare case of highly similar polymorphism.^[21]^ Both polymorphs were prepared (see the experimental procedure) and analysed (see section 2 of the Supporting Information).^[21]^ The amine group and the aliphatic chain in **2** are disordered over two positions, with an occupancy ratio of 0.7 : 0.3 in form 1, and 0.9 : 0.1 in form 2.^[21]^ The authors note that the occupancies were not affected by temperature, suggesting the presence of static positional disorder.^[21]^ As shown in Figure 2b, the chloride anion is nearby to the disorder, and participates in a ^+^N−H⋅⋅⋅Cl^−^ hydrogen bond (form 2 at 293 K: *d*_H⋅⋅⋅Cl_=2.34 Å, *d*_N⋅⋅⋅Cl_=3.02 Å, θ_N‐H⋅⋅⋅Cl_=173°), along with several close contacts.

The crystal structure of **3** was reported by McDowell and features crystallographic disorder of its two chloride anions, as shown in Figure 2c.^[22]^ The occupancy ratio reported by the authors is 1.01 : 0.49 : 0.71 for Cl(1), Cl(2), and Cl(3), respectively (see Figure 2c). The crystal structure of **3** was reported in 1980 and was solved with constraints, resulting in a flat piperazinium ring. The crystal structure was modified through structural modelling, adding the two missing hydrogen atoms to the nitrogen atoms of the piperazinium ring. These two hydrogen atoms have been labelled H(x) and H(y) for the purpose of clarity, and were placed either above or below the ring. The two chloride positions were chosen based on the positions of H(x) and H(y), maintaining the H⋅⋅⋅Cl^−^ hydrogen bond. Following a geometry optimization using DFT, the piperazinium ring became puckered and three distinct conformations were found (see Figure S15 of the Supporting Information). In conformation 1, the hydrogen atoms H(x) and H(y) were placed below and above the piperazinium ring, respectively, as shown in Figure 1. In conformation 2, the hydrogen atoms H(x) and H(y) were placed above and below the piperazinium ring, respectively. In conformation 3, the hydrogen atoms H(x) and H(y) were both placed above the piperazinium ring. A fourth conformation was attempted where both hydrogen atoms H(x) and H(y) were placed below the ring, but the optimizations failed to converge. Conformation 1 and 2 were the lowest energy conformations and are shown in Figure 2c, with conformation 3 instead having a much higher energy. As a result, focus was placed on conformations 1 and 2. Due to the disorder of the ^+^N−H hydrogen position, the chloride anion is also disordered.

### ^13^C and ^1^H Solid‐state NMR

**Figure 3:**
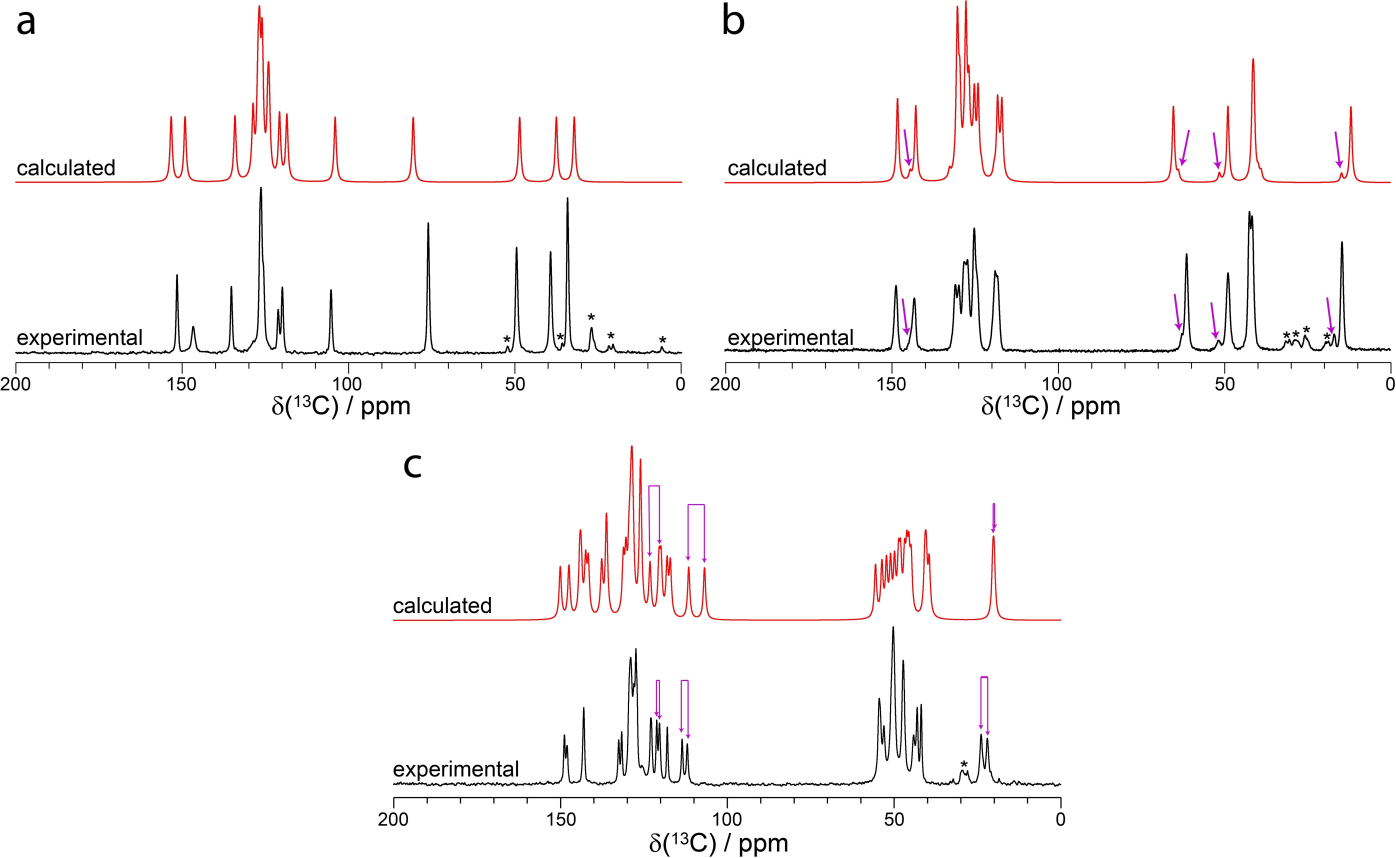
Experimental (black) and GIPAW‐calculated (red) ^1^H−^13^C solid‐state NMR CPMAS spectrum of (a) **1**, (b) **2** (form 1), and (c) **3** (ν_L_=125.8 MHz, ν_MAS_=12.5 kHz). The asterisks denote spinning sidebands, and the magenta arrow denotes resolved resonances assigned to the crystallographic disorder. See Figure S10 of the Supporting Information for the ^13^C spectrum of **2** form 2. The calculated chemical shifts of **1** were determined by averaging the calculated chemical shifts for the two thiophene conformations.

^1^H−^13^C cross‐polarization magic‐angle spinning (CPMAS) solid‐state NMR experiments provided the first line of analysis for the crystallographic disorder. In all cases, the experimental ^1^H−^13^C CPMAS spectra are supported by GIPAW‐DFT calculations performed on the DFT‐optimized crystal structure. In **1**, an experimental ^1^H−^13^C CPMAS NMR spectrum features averaging of the resonances assigned to the carbon atoms of the thiophene ring, which has been suggested by C.E. Marjo et al.^[20]^ As the DFT calculations were performed using models where the thiophene ring is in its two unique positions and does not in itself consider the dynamics, the calculated chemical shifts were averaged at a 1 : 1 ratio between the two models. The averaged ^13^C chemical shifts accurately reproduced the experimental ^13^C spectrum, as shown in Figure 3a, with minor discrepancies of the chemical shifts assigned to the thiophene group (124.2 ppm, 127.3 ppm, 128.7 ppm). These minor discrepancies are attributed to the calculations not fully accounting for the dynamics. The experimental and calculated ^13^C chemical shifts for both conformations can be found in Table S2 of the Supporting Information along with additional simulated ^13^C spectra in Figure S4. A value of *σ*_ref_ was determined for each compound by comparing the experimental ^13^C chemical shifts to the GIPAW‐calculated chemical shift, with the gradient set to unity.^[23]^ Separate values of *σ*_ref_ were used for chemical shifts above and below 100 ppm.

The ^1^H−^13^C CPMAS NMR spectrum of **2**, form 1, features shoulders adjacent to the resonances at 143.3 ppm, 61.4 ppm, 48.9 ppm, and 14.7 ppm, and are highlighted by the pink arrow in Figure 3b. These shoulders have been previously reported, and have been assigned to the disorder occurring in the amine and aliphatic groups.^[21]^ As shown in Figure S10 of the Supporting Information, the ^1^H−^13^C CPMAS NMR spectrum of **2**, form 2, appears to be highly similar to form 1, apart from some broadening. Overall, the experimental ^1^H−^13^C CPMAS spectrum of **2**, for form 1 and form 2, are in excellent agreement with a previous report.^[21]^ In addition, the minor peaks which appear as shoulders on the more intense peaks are reproduced very well by the DFT calculations, as shown in Figure 3b, supporting the assignment of the crystallographic disorder to the shoulders of select resonances.

To the best of our knowledge, **3** has not been previously investigated by ^1^H−^13^C CPMAS solid‐state NMR. As shown in Figure 3c and highlighted by the magenta arrows, a ^1^H−^13^C CPMAS solid‐state NMR spectrum of **3** features the doubling of several resonances. This doubling of resonances is most commonly observed in structures with two or more crystallographically unique molecules in the asymmetric unit (e. g. *Z*′=2), while this structure only has a single molecule in the asymmetric unit (*Z*′=1). The doubling observed here has been assigned to the occurrence of crystallographic disorder of the piperazinium ring. The DFT calculations performed on the two conformations of the piperazinium ring (see Figure 2c) reproduces these doublings, and supports the occurrence of crystallographic disorder of the piperazinium ring. Tentative assignments were made using a ^1^H−^13^C CP‐HETCOR MAS NMR spectrum (Figure S20) along with GIPAW calculations and are given in Figure S19 of the Supporting Information. All experimental and calculated chemical shifts can be found in Table S5. While the NMR calculations were also performed on conformation 3, the results were not part of the simulations in Figure 3 as it was a higher energy structure, but the simulations can be found in Figure S19 of the Supporting Information. Using the integral of the ^13^C resonances at 22 and 24 ppm in **3**, and assuming a similar cross‐polarization efficiency between conformations 1 and 2, the relative population ratio is approximately 1 : 0.9.

**Figure 4:**
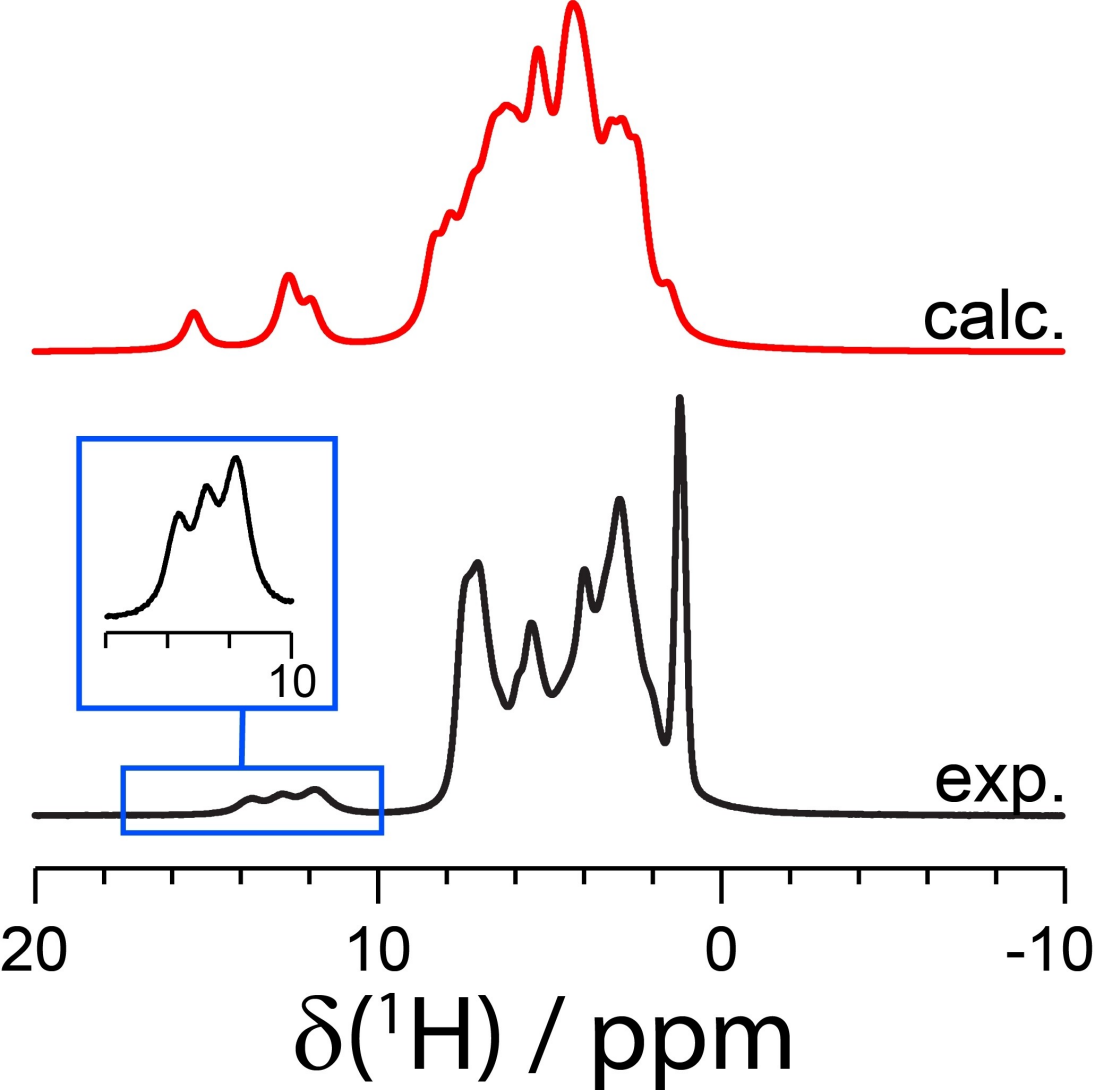
Experimental one‐pulse (black) and GIPAW‐calculated (red) ^1^H solid‐state NMR magic‐angle spinning spectrum of **3** (ν_L_=850.2 MHz, ν_MAS_=60 kHz) with background suppression. The inset in blue provides a magnified view of the N−H^+^ area.

A ^1^H one‐pulse MAS solid‐state NMR spectrum of **3**, as shown in Figure 4, further supports the occurrence of crystallographic disorder of the piperazinium ring. Three ^1^H resonances are observed experimentally (δ(^1^H)=13.6 ppm, 12.7 ppm, 11.8 ppm), which is assigned to the two protons of the piperazinium ring having more than one potential crystallographic position. As shown in the GIPAW calculations, the δ(^1^H) of the N−H hydrogen in the ^+^NH−CH_3_ moiety varies based on the two conformations of the molecule, with calculated ^1^H chemical shifts of 12.3 ppm and 14.4 ppm. The separated simulated ^1^H spectra can be found in Figure S22 of the Supporting Information, including a simulation of conformation 3. In contrast, the δ(^1^H) of the N−H hydrogen ^+^NH−R_2_R′ moiety has a difference of less than 0.1 ppm, with two resonances at chemical shifts of approximately 11.7 ppm. Overall, the three distinct ^1^H resonances observed in the spectrum supports the presence of two conformations of the piperazinium ring.

### ^35^Cl Solid‐state NMR

**Figure 5:**
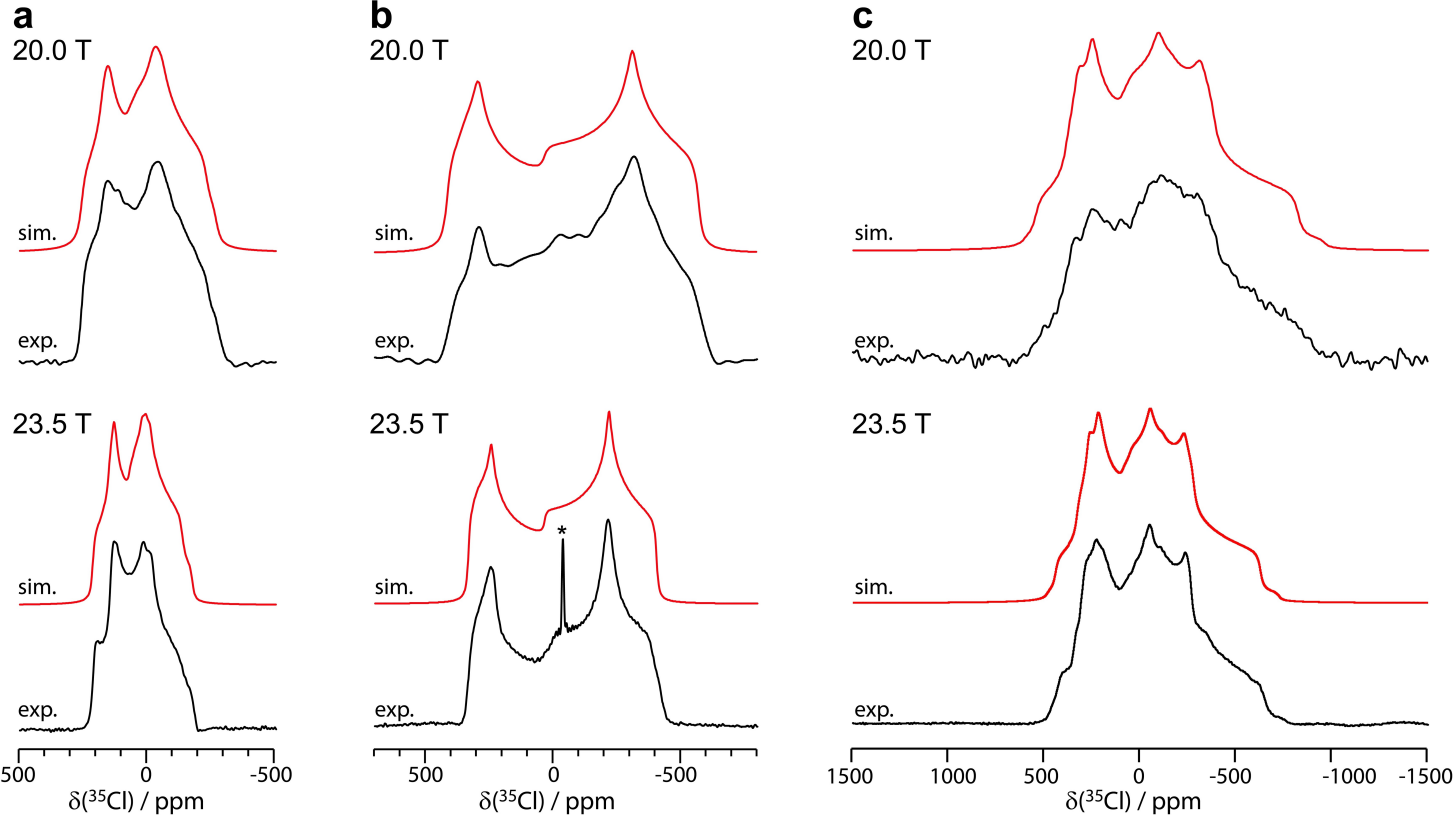
Experimental ^35^Cl solid‐state NMR spectra of a) **1**, b) **2** form 1, and c) **3** acquired at 20.0 T (above) and 23.5 T (below). The experimental spectra are shown in black, and the simulated spectra are shown in red. The asterisk above the resonance at −41.1 ppm has been assigned to a trace amount of NaCl(s) in **2**. The spectra were simulated using QUEST.

**1**, **2**, and **3** were investigated by ^35^Cl solid‐state NMR at *B*_0_ magnetic fields of 23.5 T (ν_L_(^1^H)=1 GHz), 20.0 T (ν_L_(^1^H)=850 MHz), and 11.7 T (ν_L_(^1^H)=500 MHz). The experiments were performed at multiple fields to ensure the accuracy of the spectral fits. As shown in Figure 5, the ^35^Cl NMR spectra acquired at 23.5 T yielded excellent signal intensity for each sample. When performed at 20.0 T, double‐frequency sweep (DFS)^[24]^ was used as a signal enhancement technique, and provided satisfactory spectra for **1** and **2**, while **3** lacked spectral features when compared to the spectrum acquired at 23.5 T. For the ^35^Cl spectra acquired at 11.7 T, the WURST‐QCPMG pulse sequence^[25]^ was used to increase excitation bandwidth and signal intensity, with the spectra shown in the Supporting Information (see Figure S13). Unfortunately, the ^35^Cl spectrum of **1** and **3** at 11.7 T yielded a poor signal‐to‐noise ratio. The ^35^Cl NMR parameters were obtained by analytical fitting using QUEST^[26]^ and have been summarized in Table 1. These ^35^Cl NMR parameters are within the range of previously observed parameters for pharmaceutical hydrochlorides,[^11a^, ^11g^] and are complemented by the GIPAW‐DFT calculations. Improvements to the ^35^Cl NMR calculations can potentially be made using optimized force fields.^[27]^

**Table 1:** Experimental ^35^Cl solid‐state NMR parameters,^[a]^ and GIPAW DFT calculated ^35^Cl NMR parameters (*in italics*).

Parameter	**1** (conf A)	**1** (conf B)	**2** form 1	**3** (site 1)	**3** (site 2)	**3** (site 3)
*|C*_Q_ |[MHz]	4.1±0.2	4.1±0.3	6.5±0.1	5.5±0.1	7.5±0.2	7.3±0.3
*(calculated)*	*5.81*	*5.92*	*8.07*	*9.2* ^[c]^	*8.1* ^[c]^	*8.5* ^[d]^
*η*	0.55±0.05	0.44±0.06	0.27±0.03	0.42±0.06	0.40±0.03	0.65±0.1
*(calculated)*	*0.31*	*0.34*	*0.20*	*0.41* ^[c]^	*0.26* ^[c]^	*0.59* ^[d]^
*δ*_iso_ [ppm]^[b]^	75±10	70±10	65±10	100±10	55±15	50±20
*(calculated)*	*126*	*139*	*90*	*116* ^[c]^	*72* ^[c]^	*57* ^[d]^
*Ω* [ppm]	60±30	70±40	100±20	60±30	60±30	80±30
*κ*	0.5±0.3	0.3±0.5	−0.5±0.3	0.2±0.3	0±0.3	0±1
*α* [°]	60±20	90±30	35±10	^[e]^	^[e]^	^[e]^
*β* [°]	30±10	30±20	0±10	^[e]^	^[e]^	^[e]^
*γ* [°]	70±20	90±40	20±10	^[e]^	^[e]^	^[e]^
rel. intensity	1	1		0.7±0.1	1±0.1	0.3±0.1
crystallographic assignment				Cl(2)^[f]^	Cl(1)^[f]^	Cl(3)^[f]^

[a] Spectral fit performed with QUEST. The Euler angles follow the ZYZ convention.^[26]^ [b] Experimental chemical shifts referenced using KCl(s) at 8.54 ppm. Calculated chemical shifts referenced using σ_ref_(^35^Cl)=962 ppm and $\delta_{calc}=\;\frac{\sigma_{ref}-\;\sigma_{calc}}{1-\;\sigma_{ref}}$ . [c] Obtained on conformation 1. The full list of calculated values can be found in Table S7 of the Supporting Information. [d] Obtained on conformation 2. The full list of calculated values can be found in Table S7 of the Supporting Information. [e] Significant uncertainty due to spectral overlap. [f] Tentatively assigned based on the relative intensity.

In the case of **1**, a slight broadening of the right horn from −19 ppm to 14 ppm was observed in the ^35^Cl NMR spectrum acquired at 23.5 T, and was fitted best using a two‐site model (see Figure 5a) with highly similar fitting parameters between both sites (see Table 1). Attempts at fitting the ^35^Cl NMR spectra of **1** using a single site model did not fully reproduce the line shape of the right horn between δ(^35^Cl)=10 ppm to −13 ppm (see Figure S5 of the Supporting Information). The similarities in the ^35^Cl fitting parameters between both sites can be rationalized by an averaging effect from the rotation of the thiophene group, and the highly similar crystallographic environment between both conformations, as shown in Figure 1c. Curiously, while the ^13^C resonances of the thiophene group in **1** at 11.7 T is nearly fully averaged (ν_L_(^13^C)=125.8 MHz), the ^35^Cl spectrum of **1** at 23.5 T (ν_L_(^35^Cl)=98.0 MHz) does not exhibit complete averaging. Rather, minor differences within experimental error in *δ*_iso_, *η*, *Ω*, *κ*, and the Euler angles are observed. The values of *C*_Q_ measured in **1** of 4.1±0.2 MHz and 4.1±0.3 MHz are smaller relative to those measured in **2** and **3**, perhaps as an effect of the dynamics. Interestingly, only a single site can be used to fit the ^35^Cl NMR spectrum of **1** at 20.0 T, highlighting the potential gains offered at higher magnetic fields.

In **2**, the ^35^Cl NMR experiments acquired at 23.5 T, 20.0 T, and 11.7 T each provided significant signal intensity, as can be seen in Figure 5b and Figure S13 of the Supporting Information. Form 2 was not analysed at 23.5 T due to time constraints and the highly similar spectra obtained between form 1 and form 2 at 11.7 T and 20.0 T. The data was fitted using a single site model, despite the structure exhibiting crystallographic disorder over two positions of occupancy. The horns in the ^35^Cl spectra of **2** are broader than of those observed in samples **1** and **3**, perhaps in part due to the crystallographic disorder. Unfortunately, the effect of the crystallographic disorder could not be readily observed from the ^35^Cl spectrum, and a single site model was sufficient to properly fit the spectra. Both forms of **2** were investigated by ^35^Cl NMR at 11.7 T and 20.0 T, and their spectra appeared to be superimposable (see Figure S13 of the Supporting Information), supporting that both structures are highly similar.

In the X‐ray structure of **3**, which is a dihydrochloride salt, the two chloride anions are disordered over three positions, as determined by X‐ray crystallography and confirmed by ^13^C and ^1^H solid‐state NMR. The ^35^Cl NMR spectrum acquired at 23.5 T, as shown in Figure 5c, displays characteristic spectral features that could only be fitted using a three‐site model. The relative intensity of the three sites were 1.0 : 0.7 : 0.3, which is comparable to the reported occupancies of 1.01 : 0.71 : 0.49. Small differences in the relative intensities may arise due to each ^35^Cl site having distinct *T*_1_ and *T*_2_ relaxation times. The three‐site model supports the occurrence of crystallographic disorder observed in the crystal structure, and is most reliably observed in the ^35^Cl spectrum acquired at 23.5 T. As discussed above, the disorder in **3** appears to originate from the hydrogen position on the piperazinium ring, which has been confirmed to be disordered by ^1^H solid‐state NMR, with the H⋅⋅⋅Cl^−^ hydrogen bond steering the chloride anions into several positions of occupancy. The ^35^Cl solid‐state NMR results further supports these findings, observing three unique ^35^Cl sites. As a three‐site model was used in the fitting, the parameters have larger errors than in **1** and **2**, and the Euler angles could not be reliably determined. However, the ^35^Cl fitting parameters across the three sites in **3** are distinguishable, most notably for site 1. For instance, a 50±22 ppm difference in *δ*_iso_ and 2±0.3 MHz difference in the *C*_Q_ is observed between sites 1 and 3.

## Conclusions

In conclusion, we have investigated three pharmaceutical hydrochloride salts exhibiting distinct cases of disorder by solid‐state NMR: dynamic disorder (**1**), static disorder of the amine and aliphatic carbon chain (**2**), and static disorder of the piperazinium ring and chloride anion (**3**). This crystallographic disorder has been confirmed by ^1^H and ^13^C solid‐state NMR spectroscopy and is in excellent agreement with the GIPAW calculations. ^35^Cl solid‐state NMR provided further evidence for the crystallographic disorder, with the resolution enhancements obtained at 23.5 T allowing the crystallographic disorder to be resolved in **1** and **3**. However, in the case of **2**, the ^35^Cl NMR spectra featured spectral broadening and the effect of disorder was not clearly resolved, even at high magnetic fields. Combining the data obtained from ^1^H, ^13^C, and ^35^Cl NMR, we show that **3** exhibits a unique case of disorder, whereby the ^+^N−H hydrogen positions of the piperazinium ring are disordered, steering the chloride anions into disorder over three positions of occupancy. Overall, we show that high field ^35^Cl solid‐state NMR complements conventional ^1^H and ^13^C NMR in characterizing the crystallographic disorder in pharmaceutical hydrochlorides.

## Experimental Section

Duloxetine hydrochloride (**1**), promethazine hydrochloride (**2**), and trifluoperazine dihydrochloride (**3**) were purchased from Sigma‐Aldrich. **1** and **3** were used without further purification. The two polymorphs of **2** (form 1, form 2) were prepared in a powdered form following the experimental procedure of Borodi et al.^[21]^ Form 1 was prepared by stirring 500 mg of promethazine hydrochloride in tetrahydrofuran at room temperature for 4 hours and filtering the powder. Hexane was added to the remaining solution, collecting the precipitate. Form 2 was prepared by stirring 500 mg of promethazine hydrochloride in acetonitrile for 4 hours and filtering the solution. The products were left to dry overnight and were used without further manipulations. Powder X‐ray diffractions were performed on a Bruker D4 Endeavor diffractometer, scanning 2θ from 5° to 65° with steps of 0.02° at a rate of 5°/minute (Cu Kα1/2=1.5418 Å). All powder X‐ray diffraction data can be found in the Supporting Information.

^*13*^ *C Solid‐state NMR*. All samples were packed into 4 mm zirconium oxide MAS rotors. Experiments were performed on either a Bruker Avance III spectrometer operating at a ^1^H Larmor frequency of 500 MHz using a 4 mm Bruker HXY probe. A MAS rate of 12.5 kHz was used throughout all experiments. The 1D ^13^C CPMAS spectra were acquired using a ramped contact pulse from 50 % to 100 % on the ^1^H channel,^[28]^ a contact time of 2 ms, a ^1^H π/2 pulse duration of 2.5 μs, a 3 s recycle delay, co‐adding 1024 transients, and using SPINAL64 proton decoupling^[29]^ with a ^1^H nutation frequency of 100 kHz and ^1^H π‐pulses of 3.8 μs. The ^13^C spectra were calibrated using the carbonyl resonance of *L*‐alanine and referenced to 178.8 ppm relative to adamantane at 38.52 ppm.^[30]^

^*1*^ *H solid‐state NMR*. All samples were packed into 1.3 mm zirconium oxide MAS rotors. Experiments were performed on a Bruker Avance NEO spectrometer operating at a Larmor frequency of 850.2 MHz, using a X/Y/H–F 1.3 mm probe at 60 kHz MAS in double resonance mode. A ^1^H 90° pulse duration of 2.5 μs was used, corresponding to a ^1^H nutation frequency of 100 kHz. The one‐dimensional spectrum was acquired with background suppression. A recycle delay of 2 s was used for **3**. 32 transients were co‐added. The ^1^H spectra were referenced using the CH_3_ resonance of *L*‐alanine to 1.1 ppm, relative to adamantane at 1.85 ppm.^[30b]^

^*35*^ *Cl solid‐state NMR*. ^35^Cl NMR experiments were performed at 11.7 T (ν_L_(^1^H)=500 MHz) on a Bruker Avance III NMR spectrometer using a Bruker 7 mm HX MAS probe. A WURST‐QCPMG pulse sequence^[31]^ was used with 50 ms pulses swept over 1 MHz, a 5000 Hz spikelet separation, a 2 s recycle delay, and co‐adding 11936 transients (**2**, form 1) or 18192 transients (**2**, form 2) with continuous wave ^1^H decoupling. Experiments were repeated at 20.0 T (ν_L_(^1^H)=850.2 MHz) on a Bruker Avance NEO spectrometer using a 4 mm probe, and samples packed into 4 mm zirconium oxide rotors. A double frequency sweep^[24]^ was applied with a nutation frequency of 32 kHz for 2 ms, followed by a spin echo using a ^35^Cl 90° pulse duration of 3 μs, corresponding to a ^35^Cl nutation frequency of 83.3 kHz, and a recycle delay of 0.5 s. Lastly, static ^35^Cl NMR experiments were performed at 23.5 T (ν_L_(^1^H)=1 GHz) on a Bruker Avance NEO spectrometer using a Bruker 7 mm X MAS probe and samples packed in 7 mm zirconium oxide rotors. A quadrupolar echo (π/2‐τ‐π/2‐aq) was applied using a ^35^Cl 90° pulse duration of 4 μs, corresponding to a ^35^Cl nutation frequency of 62.5 kHz. A recycle delay of 0.5 s were used for each sample. In all cases, the ^35^Cl chemical shifts were referenced to KCl(s) at 8.54 ppm,^[32]^ and the ^35^Cl spectra were fit using QUEST for exact simulation.^[26]^

*NMR calculations*. All DFT^[33]^ calculations were performed using the gauge‐including projector augmented‐wave (GIPAW)^[34]^ method as implemented in CASTEP^[35]^ as part of Materials Studio version 17.^[36]^ The crystal structures obtained from the experimental X‐ray crystallography results were used as the structural models for the calculations. The structures were optimized by DFT to allow all bond lengths and atom positions to relax. For **1** and **2**, two input models were generated by splitting the disorder into their two respective positions. For **3**, multiple input structures were generated by adding the ^+^N−H proton either above or below the piperazinium ring. The GGA PBE functional^[37]^ was employed for all calculations, beginning with a geometry optimization prior to calculating the NMR chemical shifts. The geometry optimization was performed with TS DFT‐D correction,^[38]^ on‐the‐fly ultrasoft pseudopotentials, and Koelling‐Harmon relativistic treatment. The cutoff energy was 600 eV and the *k*‐point separation was 0.05 Å^−1^. NMR calculations were subsequently performed using the same parameters as the geometry optimization, but with a cutoff energy of 700 eV. The calculated σ_iso_ values were extracted and converted into δ_iso_ (see the Supporting Information for the values of σ_ref_) using the Magres2Topspin script.^[23]^

## Supporting Information Summary

The supplementary information contains powder X‐ray diffraction data and additional experimental solid‐state NMR and GIPAW calculated data.

Additional references cited within the Supporting Information.^[39]^

## Author Contributions

The manuscript was written through contributions of all authors. All authors have given approval to the final version of the manuscript.

## Conflict of interest

The authors declare no conflict of interest.

1

## Supporting information

As a service to our authors and readers, this journal provides supporting information supplied by the authors. Such materials are peer reviewed and may be re‐organized for online delivery, but are not copy‐edited or typeset. Technical support issues arising from supporting information (other than missing files) should be addressed to the authors.

Supporting Information

## Data Availability

Data will be made available upon acceptance at wrap.warwick.ac.uk.
