# Supplementary material for: Exploring the Potential of Multinuclear Solid‐State 1H, 13C, and 35Cl Magnetic Resonance To Characterize Static and Dynamic Disorder in Pharmaceutical Hydrochlorides
Source: Chemphyschem. 2022 Nov 7;24(3):e202200558. doi: 10.1002/cphc.202200558 (PMC10099218; doi:10.1002/cphc.202200558)
Supplement: Supplementary file 1 — Supporting Information [file CPHC-24-0-s001.pdf]

ChemPhysChem

Supporting Information

Exploring the Potential of Multinuclear Solid-State ^{1}H, ^{13}C, and ^{35}Cl Magnetic Resonance To Characterize Static and Dynamic Disorder in Pharmaceutical Hydrochlorides

Patrick M. J. Szell, Zainab Rehman, Ben P. Tatman, Leslie P. Hughes,* Helen Blade, and Steven P. Brown*

Table of Contents

Summary of the X-ray Crystal Structures. S2
1.0 - Duloxetine hydrochloride (1). S3
1.1 - X-ray Crystallography. S3
1.2 - Powder X-ray Diffraction. S3
1.3 – ^{13}C Solid-State NMR. S4
1.4 – ^{35}Cl Solid-State NMR. S7
2.0 – Promethazine hydrochloride (2). S8
2.1 – X-ray Crystallography. S8
2.2 - Powder X-ray Diffraction. S8
2.3 – ^{13}C Solid-State NMR S10
2.4 – ^{35}Cl Solid-state NMR. S13
3.0 – Trifluoperazine dihydrochloride. S14
3.1 – X-ray Crystallography S14
3.2 – Geometry Optimization S15
3.3 – Powder X-ray Diffraction S17
3.4 – ^{13}C Solid-State NMR S18
3.5 – ^{1}H Solid-State NMR S22
3.6 – ^{35}Cl Solid-State NMR S24

Summary of the X-ray Crystal Structures.

Table S1. Summary of the X-ray crystal structures used in this study.

compound	**1**	**2** (form 1)[a]	**2** (form 2)[a]	**3**
year	2009	2012	2012	1980
CSD reference #	747361	919111	919112	1270274
author(s)	Bhadbhade et al.[1]	Borodi et al.[2]	Borodi et al.[2]	McDowell[3]
Z	2	4	4	8
acquisition temp. (K)	150(2)	293(2)	293(2)	295

[a] Multiple structures reported at distinct temperatures.

1.0 - Duloxetine hydrochloride (1).

1.1 - X-ray Crystallography.

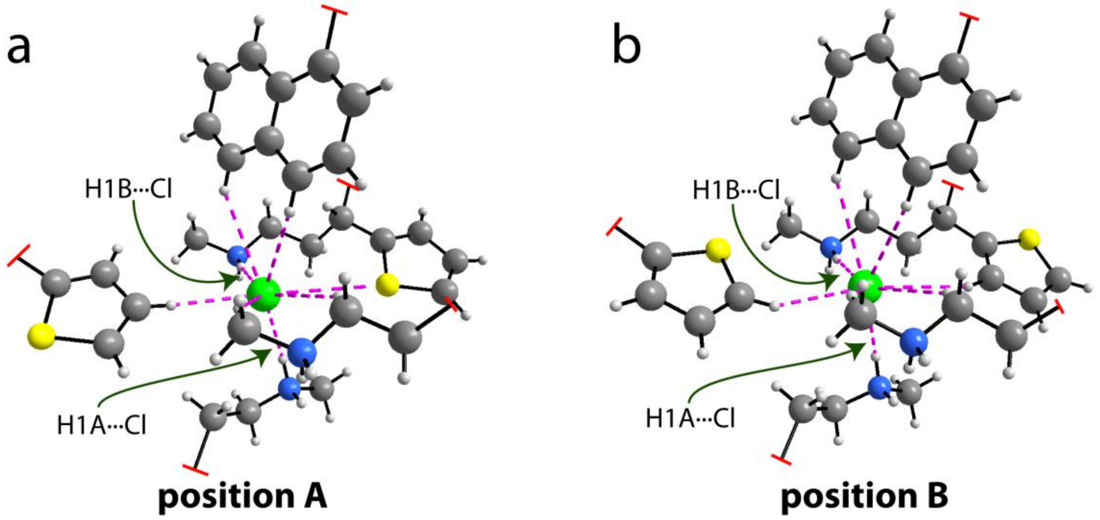

Figure S1. Coordination sphere surrounding the chloride anion in **1**, showing the two thiophene orientations: (a) position A, and (b) position B. The red bars denote the remainder of molecules that were removed for clarity, and the dashed magenta lines highlight all nearby contacts surrounding the Cl^- anion within 3.2 Å for $H\cdots Cl^-$ contacts and 3.7 Å for $S\cdots Cl^-$ contacts.

1.2 - Powder X-ray Diffraction.

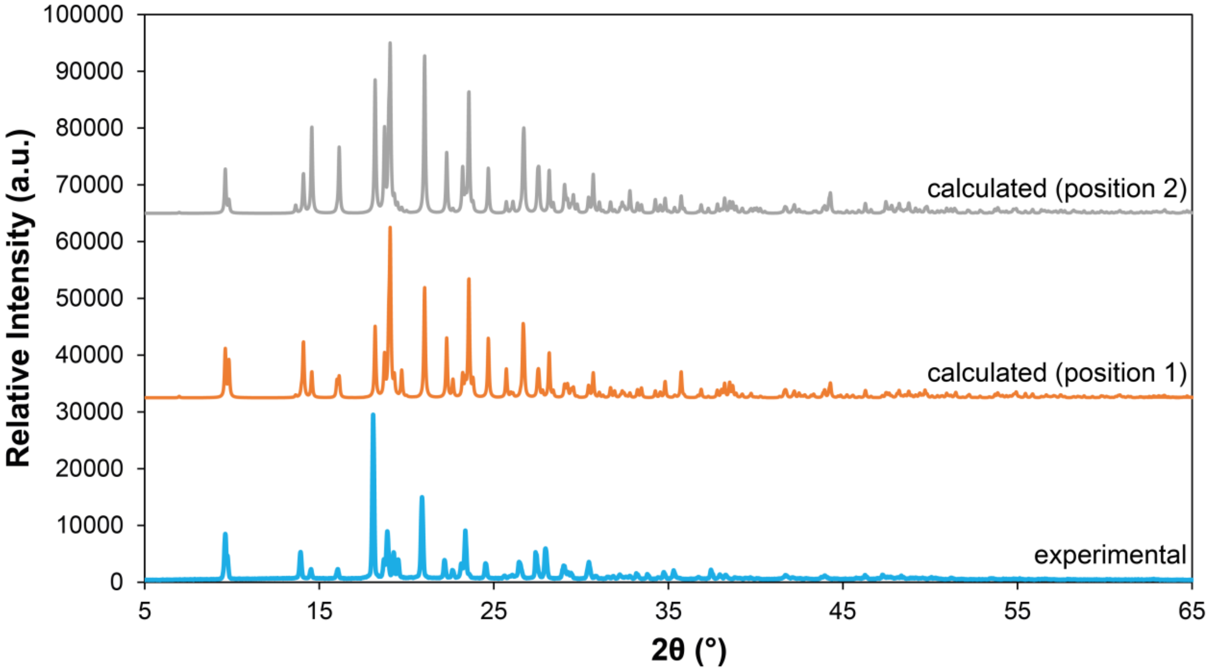

Figure S2. Experimental and calculated powder X-ray diffraction of **1**. The powder diffractograms were calculated for the experimental X-ray structure where the disorder has been separated into two separate structures. Position 1 refers to the thiophene group atom positions S1A, C13A, C14A, and C15A, whereas position 2 refers to the atom positions S1B,

C13B, C14B, and C15B. The crystal structure of 1 (CSD refcode MUCDUK) was used to calculate the theoretical diffractogram.[1]

1.3 – ^{13}C Solid-State NMR.

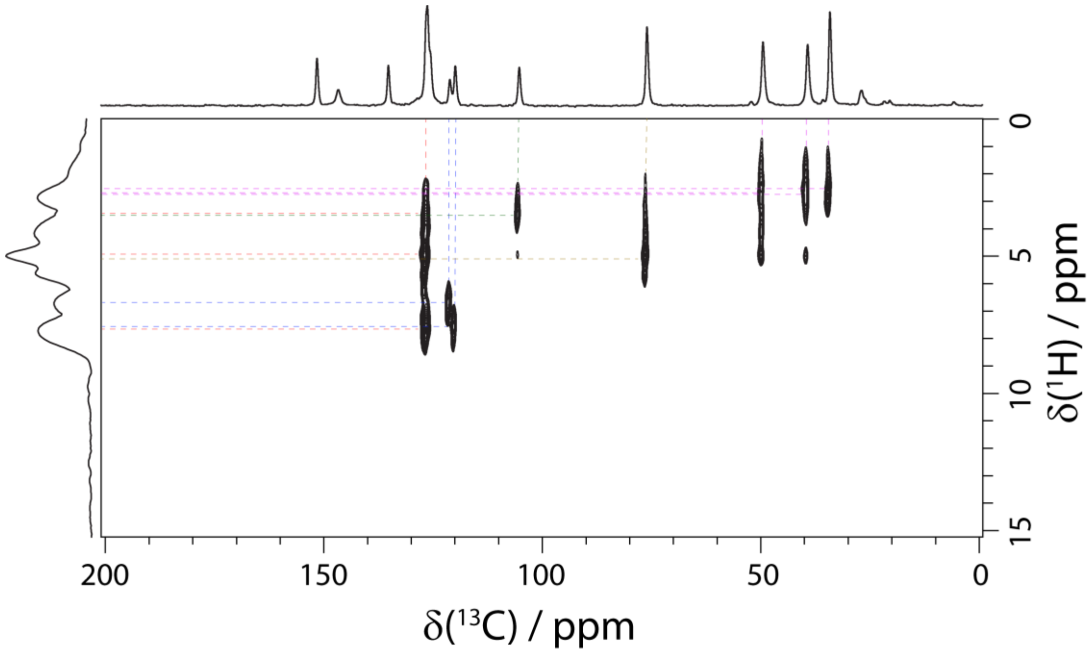

Figure S3. A ^{1}H-^{13}C CP-HETCOR solid-state MAS NMR spectrum with ^{1}H FSLG decoupling of duloxetine hydrochloride (**1**, contact time = 250 μs, ν_L = 500 MHz, ν_{MAS} = 12.5 kHz). The dashed lines are added as a guide. The horizontal axis displays an experimental ^{13}C CPMAS spectrum, while the vertical axis is a skyline projection. 36 transients were collected per t_1 FID, acquiring 192 FIDs in the indirect dimension and using the States-TPPI acquisition mode.

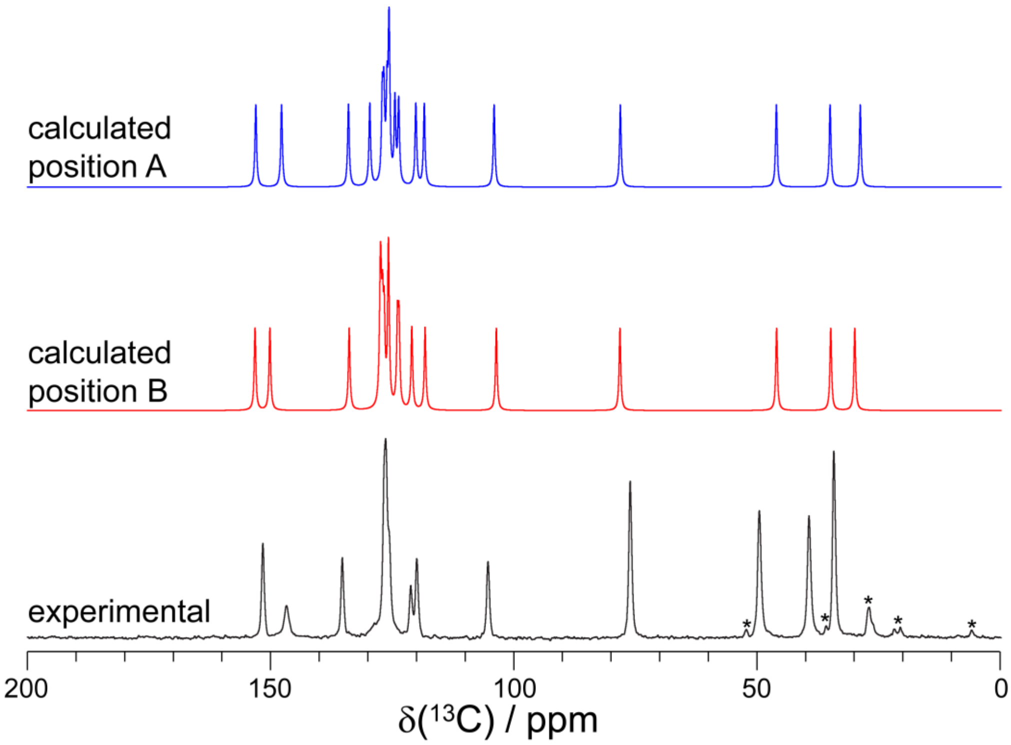

Figure S4. Experimental (black) and GIPAW-DFT calculated (red, blue) ^{1}H-^{13}C NMR CPMAS spectrum (ν_L = 125.8 MHz, ν_{MAS} = 12.5 kHz) of **1**. The asterisks denote spinning sidebands.

Table S2. Experimental and GIPAW-DFT calculated[a] ^{13}C chemical shifts of **1**. The GIPAW calculations were performed on **1** with atoms in either position A or in position B (see Figure S1).

atom label	experimental $\delta_{iso}(^{13}C)$	calculated $\delta_{iso}(^{13}C)$ position A	calculated $\delta_{iso}(^{13}C)$ position B
C9	151.5	153.3	153.3
C12	146.7	148.1	150.2
C5	135.2	134.2	134.1
C14A/B	126.3[b]	129.8	127.6
C7	126.6[b]	127.1	126.7
C13A/B	126.3[b]	126.8	127.8
C2	126.3[b]	126.3	127.1
C4	126.3[b]	126.1	126.0
C3	126.3[b]	125.9	125.9
C15A/B	126.3[b]	124.5	123.9
C10	125.6[b]	124.0	123.8
C1	121.1	120.4	121.1
C6	119.9	118.7	118.4
C8	105.2	104.3	103.7
C11	76.0	80.7	80.4
C17	49.4	48.7	48.4
C16	39.2	37.7	37.4
C18	34.1	31.8	32.5

[a] σ_{calc} converted to δ_{calc} using $\delta_{calc} = \sigma_{ref} - \sigma_{calc}$ where $\sigma_{ref}(^{13}C)$ = 170.3 ppm for chemical shifts above 100 ppm, and $\sigma_{ref}(^{13}C)$ = 172.7 ppm for chemical shifts below 100 ppm.[4]
[b] Tentative assignment.

1.4 – ^{35}Cl Solid-State NMR.

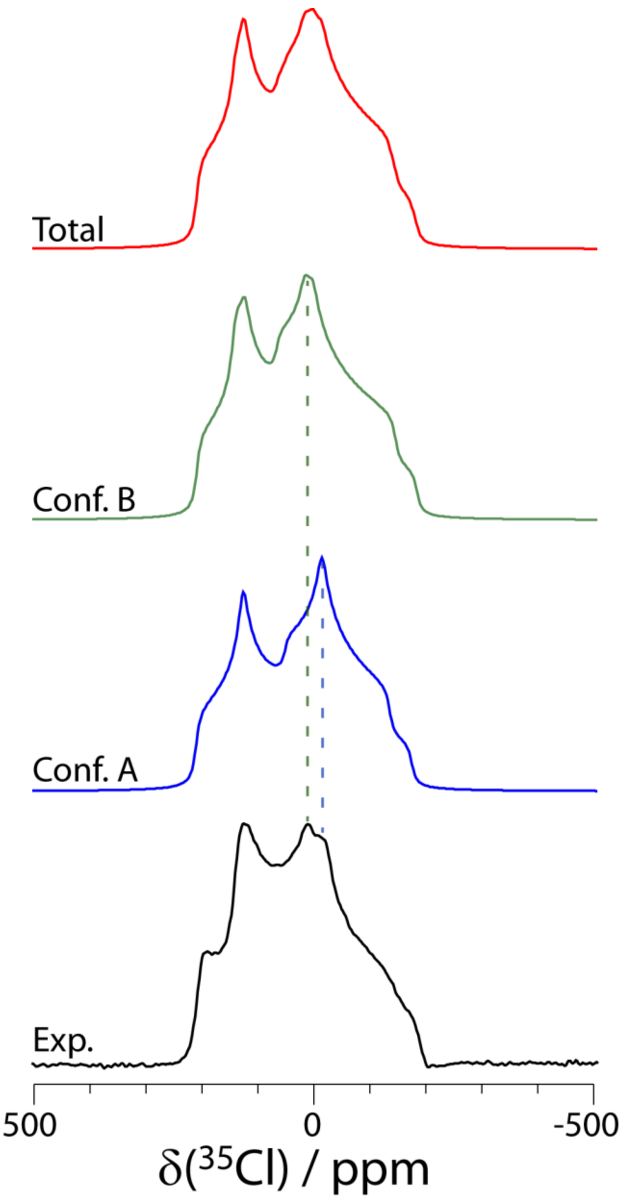

Figure S5. Experimental (black) and simulated ^{35}Cl solid-state NMR spectrum of **1** (B_0 = 23.5 T). The simulated ^{35}Cl spectrum uses the NMR parameters for conformation A (Conf. A, blue) and for conformation B (Conf. B, green), and is presented as a sum of both (Total, red). The spectral simulations were performed using QUEST[5] with the parameters from Table 1 of the main text.

2.0 – Promethazine hydrochloride (2).

2.1 – X-ray Crystallography.

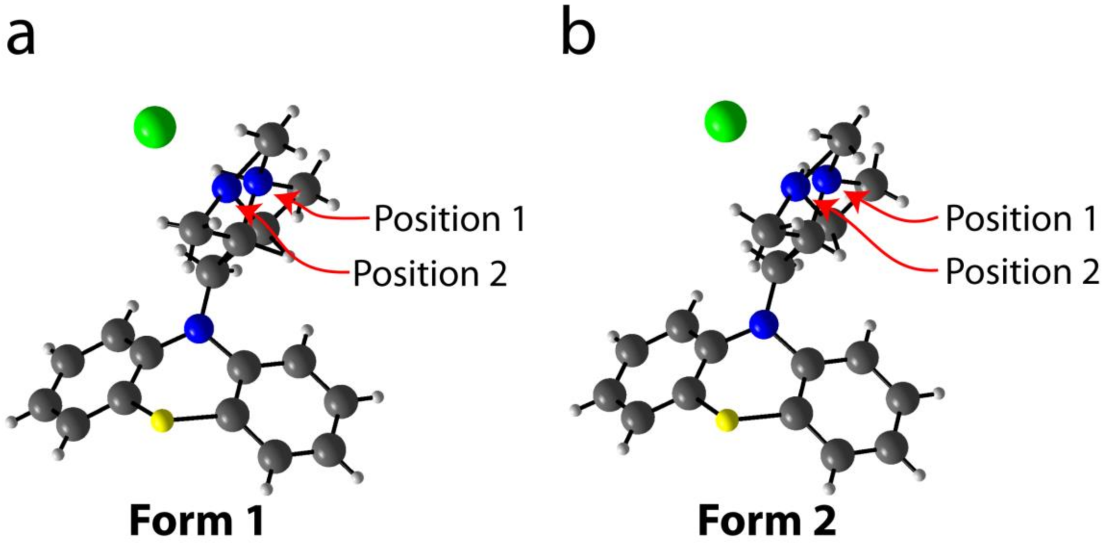

Figure S6. Depiction of the X-ray crystal structures of promethazine hydrochloride, **2**, obtained at 293(2) K, showing: (a) Form 1 (CSD# 919111), and (b) Form 2 (CSD# 919112). The two positions of occupancy are labelled: Position 1 (major position), and Position 2 (minor position). The crystal structures were reported by Borodi et al.[2]

2.2 - Powder X-ray Diffraction.

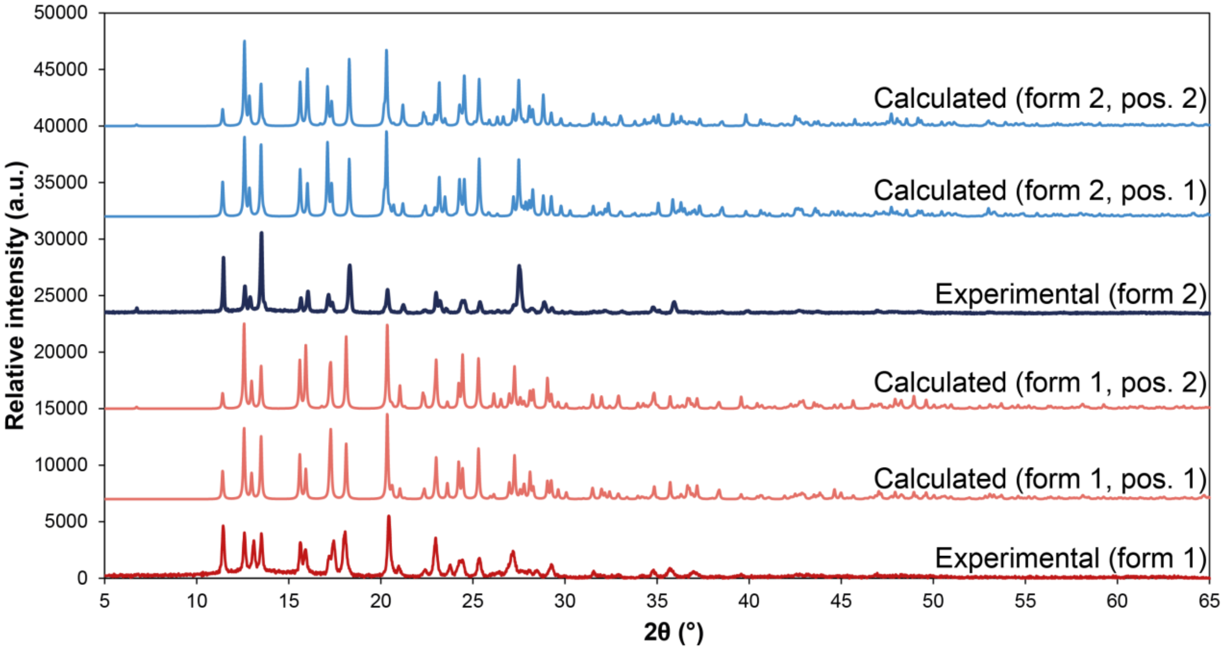

Figure S7. Experimental and calculated powder X-ray diffraction of **2**, form 1 (red traces), and form 2 (blue traces). The powder diffractograms were calculated on the DFT-optimized structures where the disorder has been separated into two separate structures. In form 1 and

form 2, position 1 refers to carbon positions C14A, C15, C16, and C17, whereas position 2 refers to carbon positions C14, C15, C16, and C17. The crystal structure of **2** form 1 (CSD refcode EAPTZC01) and **2** form 2 (CSD refcode EAPTZC02) were used to calculate the theoretical diffractograms.[2]

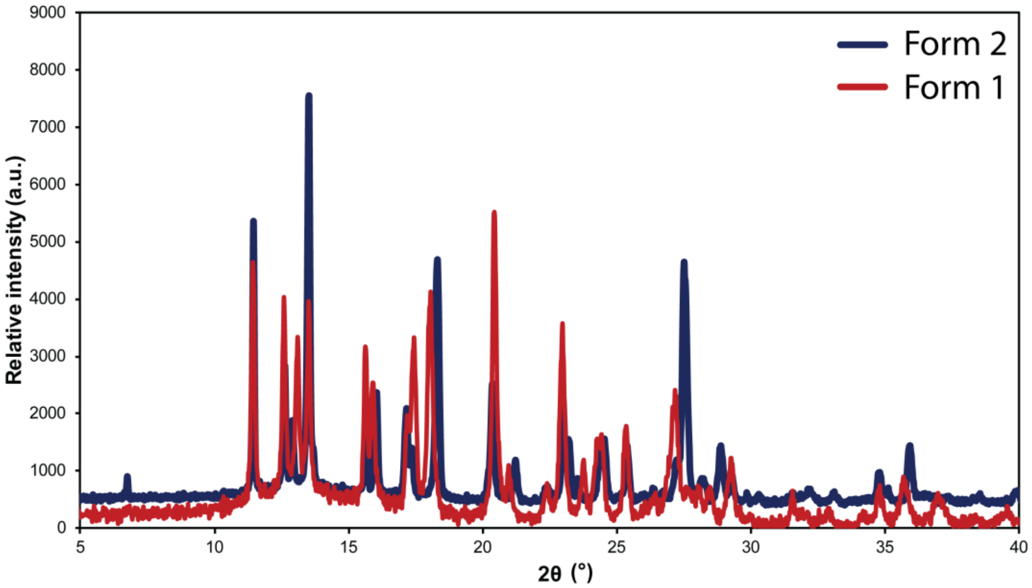

Figure S8. Experimental powder X-ray diffraction of **2**, form 1 (red traces), and form 2 (blue traces).

2.3 – ^{13}C Solid-State NMR

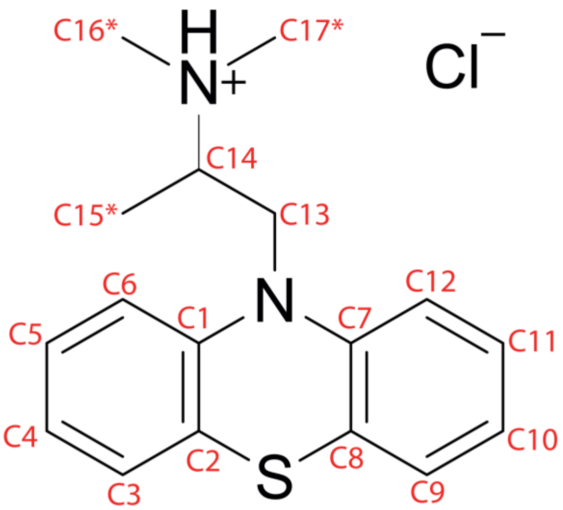

Figure S9. Molecular structure of promethazine hydrochloride (**2**) showing the carbon atom labels.

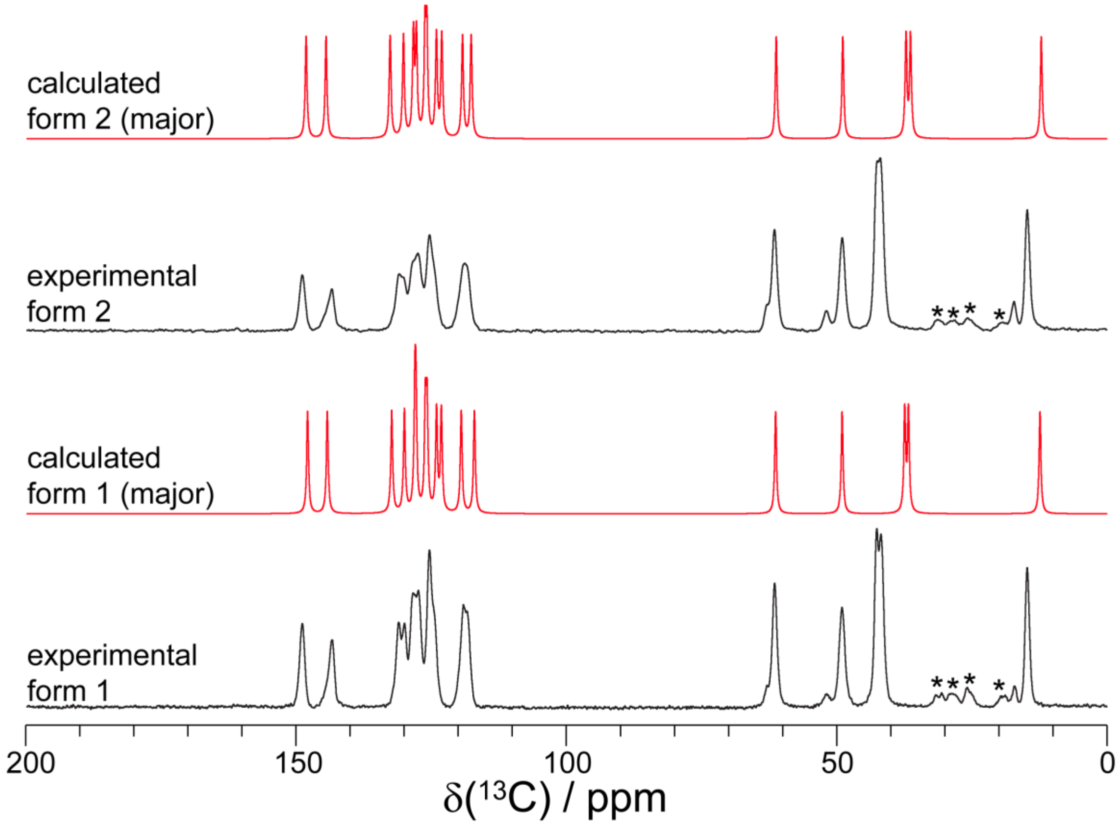

Figure S10. Experimental (black) and GIPAW-DFT calculated (red) ^{1}H-^{13}C NMR CPMAS spectrum (ν_L = 125.8 MHz, ν_{MAS} = 12.5 kHz) of **2**, form 1 (below), and form 2 (above). The asterisks denote spinning sidebands.

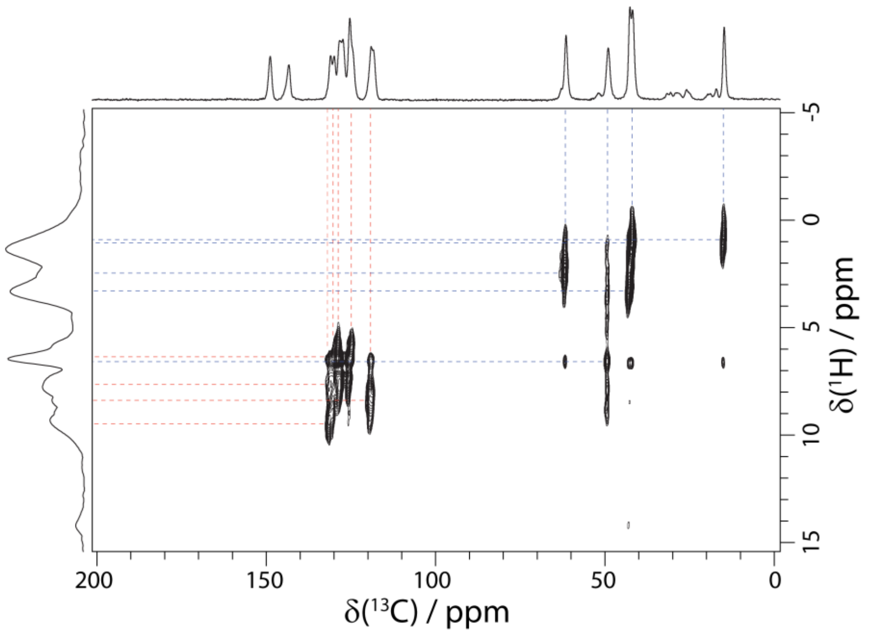

Figure S11. A ^{1}H-^{13}C CP-HETCOR solid-state MAS NMR spectrum with ^{1}H FSLG decoupling of promethazine hydrochloride form 1 (**2**, contact time = 250 μs, ν_L = 500 MHz, ν_{MAS} = 12.5 kHz). The dashed lines are added as a guide. The horizontal axis displays an experimental ^{13}C CPMAS spectrum, while the vertical axis is a skyline projection. 36 transients were collected per t_1 FID, acquiring 192 FIDs in the indirect dimension and using the States-TPPI acquisition mode.

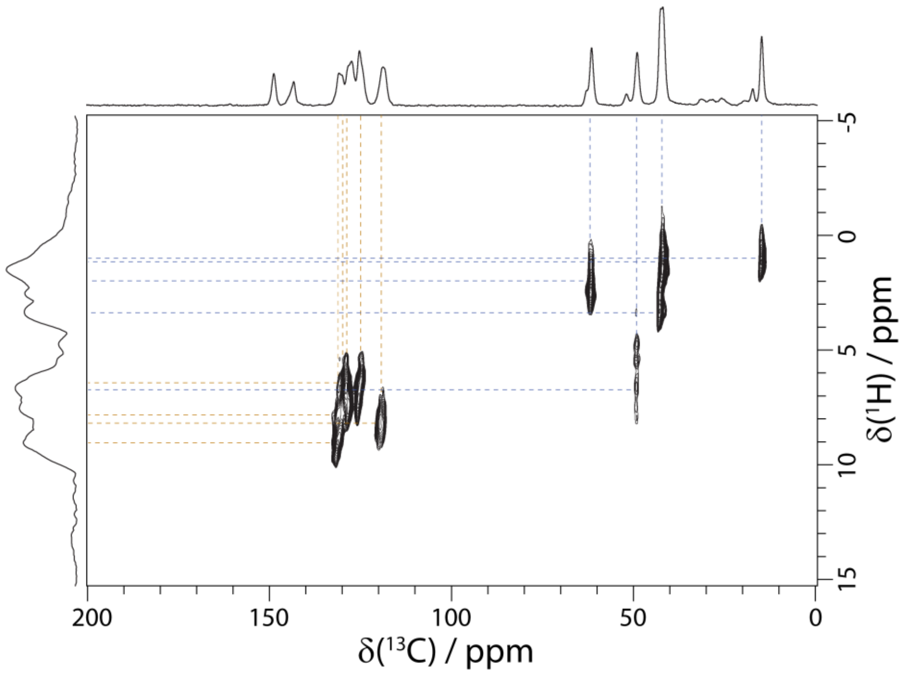

Figure S12. A ^{1}H-^{13}C CP-HETCOR solid-state MAS NMR spectrum with ^{1}H FSLG decoupling of promethazine hydrochloride form 2 (**2**, contact time = 250 μs, ν_L = 500 MHz, ν_{MAS} = 12.5 kHz). The dashed lines are added as a guide. The horizontal axis displays an experimental ^{13}C CPMAS spectrum, while the vertical axis is a skyline projection. 36 transients were collected per t_1 FID, acquiring 192 FIDs in the indirect dimension and using the States-TPPI acquisition mode.

Table S3. Experimental and GIPAW-DFT calculated[a] ^{13}C chemical shifts of **2**. The GIPAW calculations were performed on **2** with atoms in either the major or minor position of occupancy.

atom label	experimental $\delta_{iso}(^{13}C)$	calculated $\delta_{iso}(^{13}C)$ (major)	calculated $\delta_{iso}(^{13}C)$ (minor)
C1	148.8	148.4	148.3
C7	143.3	142.9	144.6
C5	130.9	130.4	132.7
C2	129.9	130.3	130.2
C11	128.3	129.6	128.4
C9	128.0	127.8	127.9
C8	127.4	127.7	126.3
C3	125.2	126.9	125.9
C4	125.2	125.2	124.2
C10	124.4	124.1	123.2
C6	118.8	118.3	119.3
C12	118.4	117.0	117.7
C14 / C14A	61.4 / 62.8[b]	65.4	63.9
C13	48.9 / 52.0[b]	49.0	51.6
C17	42.4	41.6	39.9
C16	41.9	41.2	39.1
C15	14.7 / 17.0[b]	12.0	14.9

[a] σ_{calc} converted to δ_{calc} using $\delta_{calc} = \sigma_{ref} - \sigma_{calc}$ where $\sigma_{ref}(^{13}C)$ = 170.2 ppm for chemical shifts above 100 ppm, and $\sigma_{ref}(^{13}C)$ = 173.3 ppm for chemical shifts below 100 ppm.[4]
[b] Minor peak (~5:1 intensity ratio) assigned to the minor position of occupancy.

2.4 – ^{35}Cl Solid-state NMR.

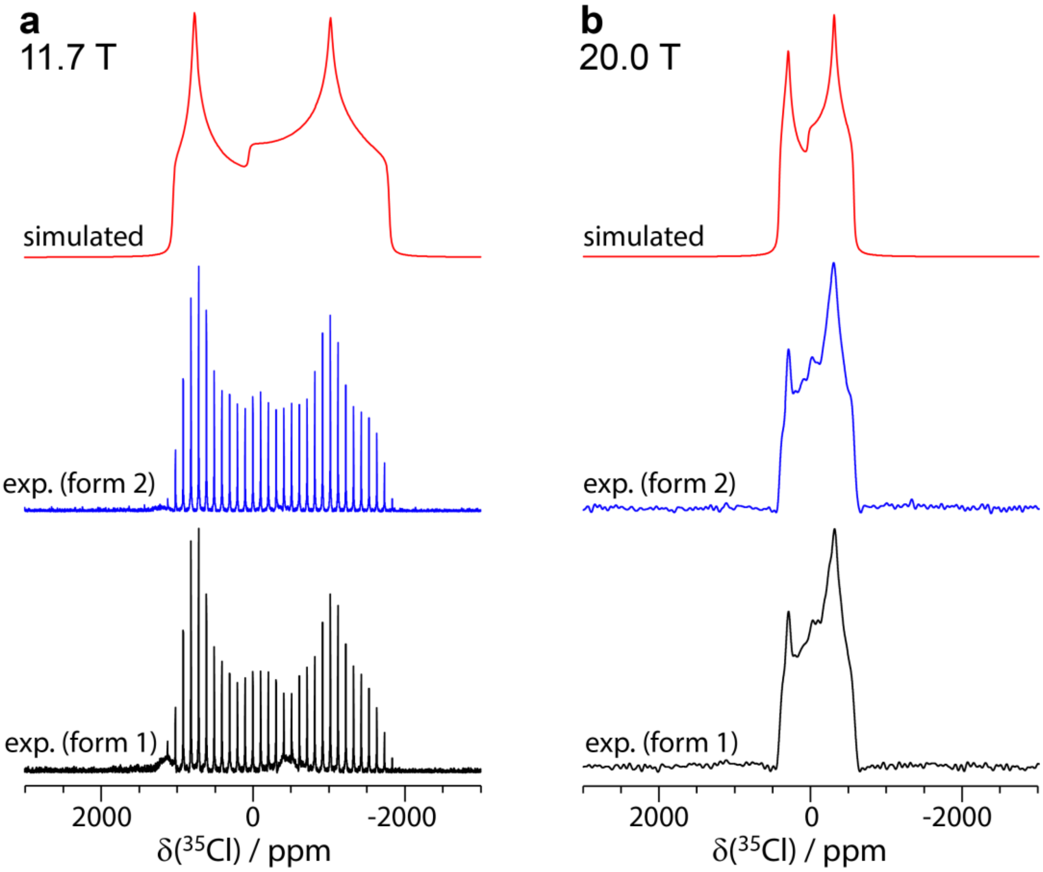

Figure S13. Experimental ^{35}Cl solid-state NMR spectrum of **2** (form **1** in black, and form **2** in blue)**,** acquired at fields of (**a**) B_0 = 11.7 T (WURST-QCPMG), and (**b**) B_0 = 20.0 T (DFS-enhanced quadrupolar echo). The simulated spectrum obtained from spectral fitting is shown in red.

Table S4. GIPAW-DFT calculated ^{35}Cl parameters for **2** form 1 and form 2 in its two disordered positions.

	**form 1**		**form 2**	
parameter	major position	minor position	major position	minor position
$\mid C_Q$ / MHz $\mid$	8.07	7.79	8.17	7.69
η	0.20	0.31	0.21	0.33
δ_{calc} / ppm[a]	90	93	93	89

[a] Calculated chemical shifts referenced using $\sigma_{ref}(^{35}Cl)$ = 962 ppm and $\delta_{calc} = \frac{\sigma_{ref} - \sigma_{calc}}{1 - \sigma_{ref}}$.

3.0 – Trifluoperazine dihydrochloride.

3.1 – X-ray Crystallography

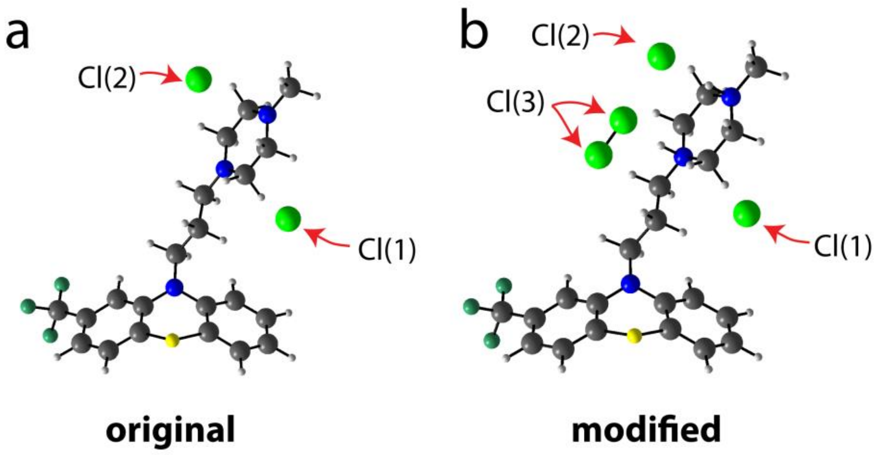

Figure S14. Depiction of the X-ray crystal structures of trifluoperazine dihydrochloride, **3**, obtained at 295 K, showing: (a) the original unmodified crystal structure as found in the CSD, and (b) the modified crystal structure adding atom Cl(3) as per the report by McDowell.[3] The three chloride positions are labelled.

3.2 – Geometry Optimization

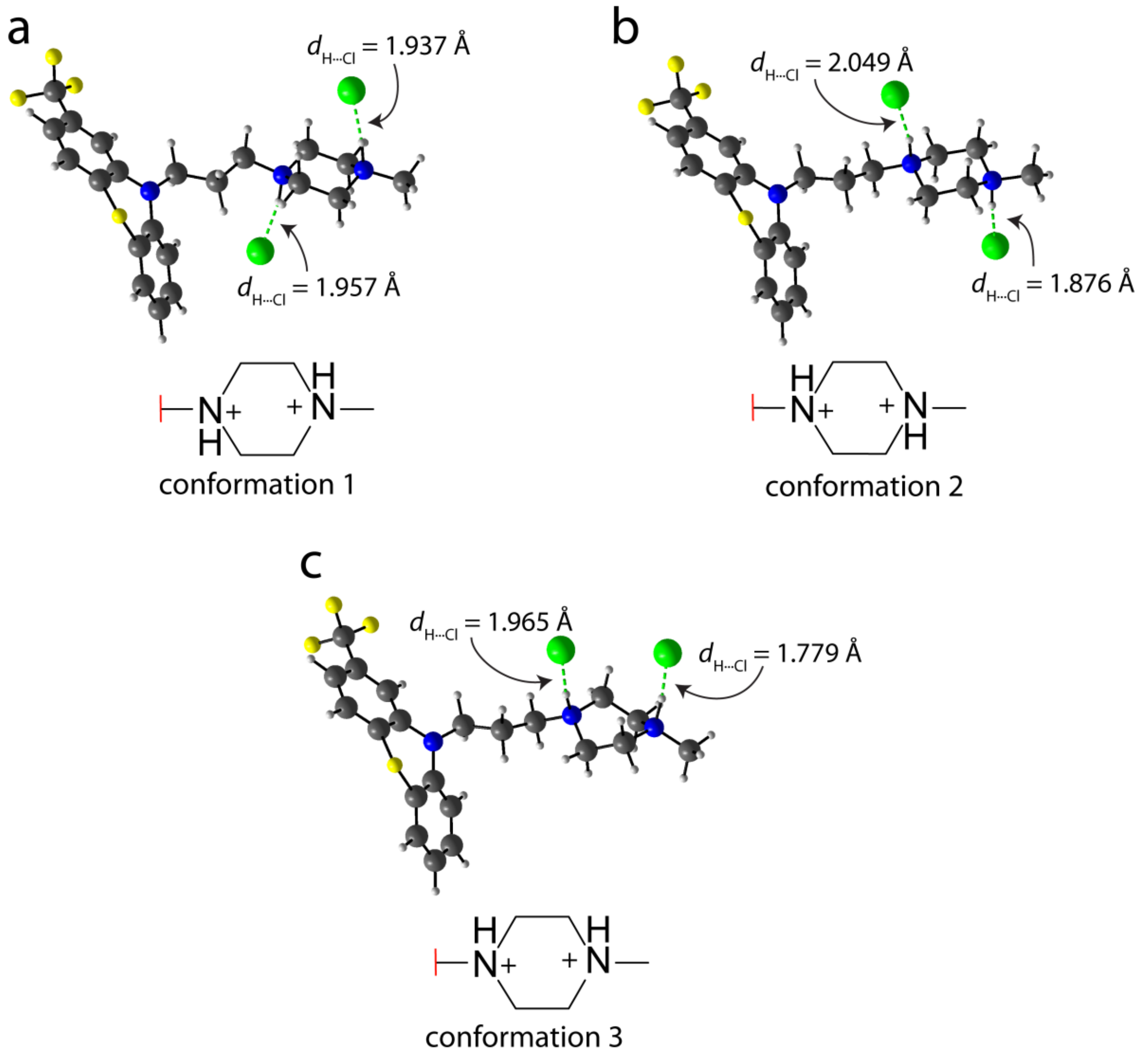

Figure S15. Depiction of the DFT-optimized structures of **3** when the piperazinium group is in (a) conformation 1 (puckered down-up), (b) conformation 2 (puckered up-down), and (c) conformation 3 (puckered up-up). The molecular structure of the piperazinium ring is given below for clarity, and the H···Cl⁻ hydrogen bond lengths are shown. The models are based on the crystal structure TFPRZC.[3] The convention up/down used here refers to the position of the hydrogen atom relative to the piperazinium ring when the molecule is placed in this orientation.

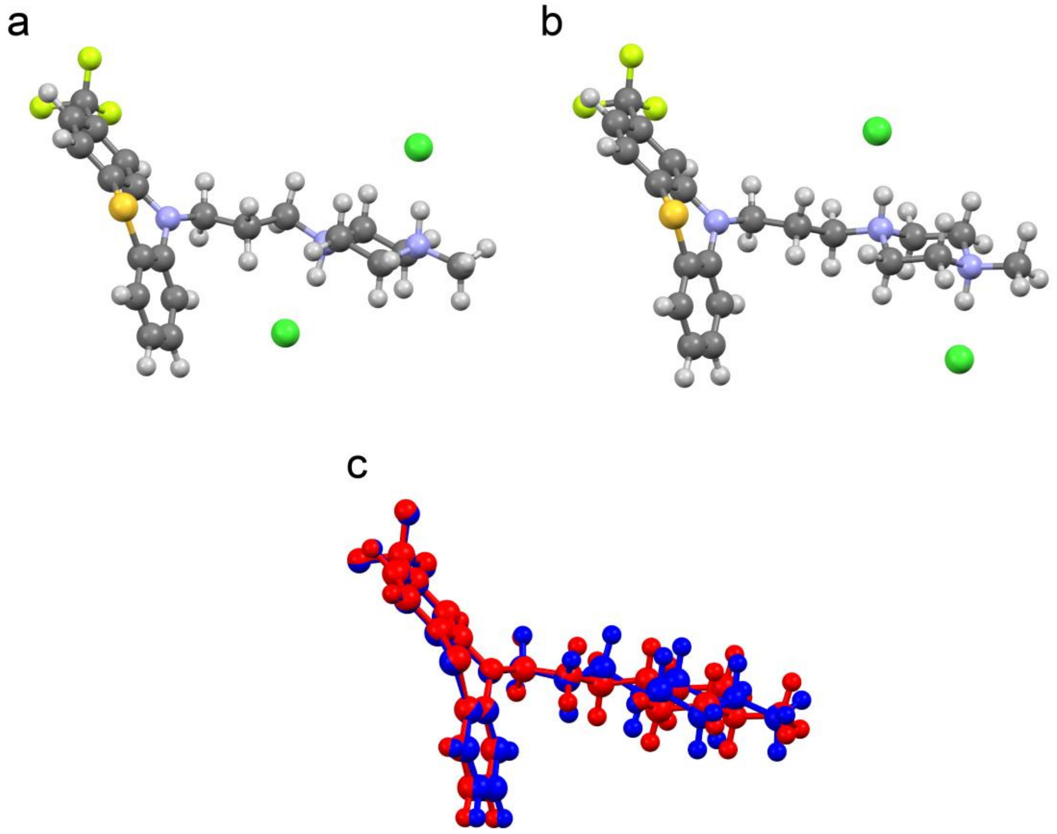

Figure S16. Depiction of the DFT-optimized structures of **3** when the piperazinium group is in (a) conformation 1 and (b) conformation 2. The overlay between conformation 1 (blue) and conformation 2 (red) is shown in (c). The models are based on the crystal structure TFPRZC.[3]

3.3 – Powder X-ray Diffraction

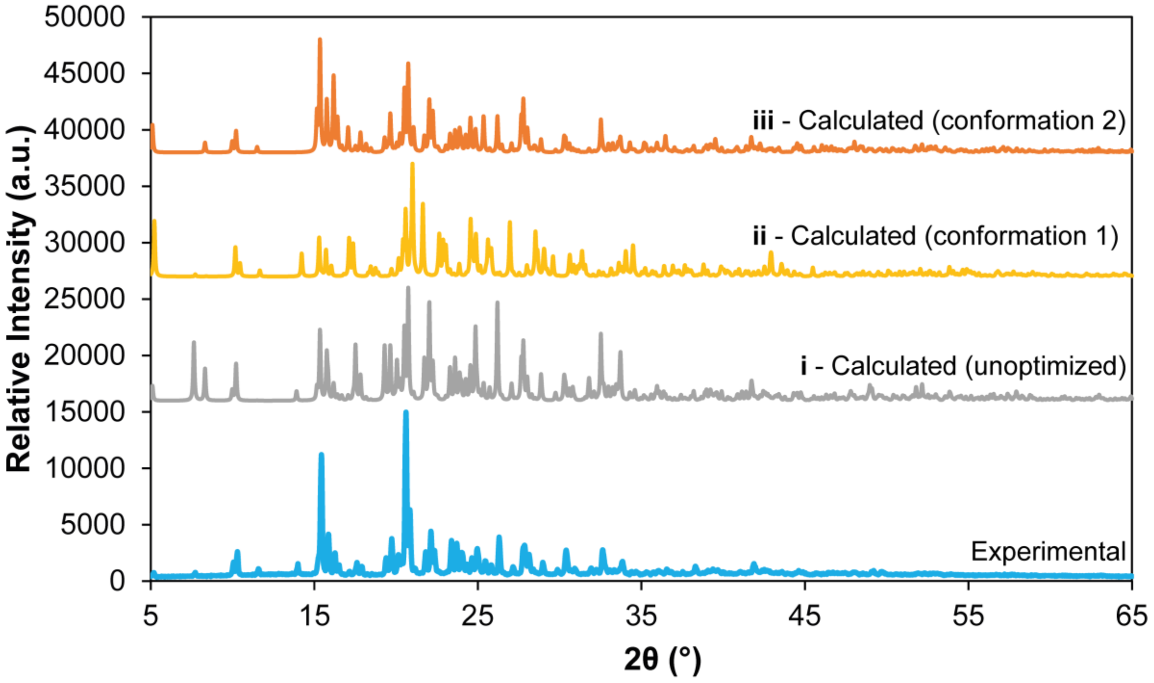

Figure S17. Experimental and calculated powder X-ray diffraction of **3**. The theoretical powder diffractograms were calculated based on the crystal structure TFPRZC (CSD refcode).[3] The diffractogram denoted by **i** was calculated using the unoptimized crystal structure featuring all three chlorine positions. The diffractogram denoted by **ii** was calculated using the optimized crystal structure with the piperazinium ring being in conformation 1 (see Figure S15), and **iii** was calculated using the optimized crystal structure with the piperazinium ring being in conformation 2 (see Figure S15).

3.4 – ^{13}C Solid-State NMR

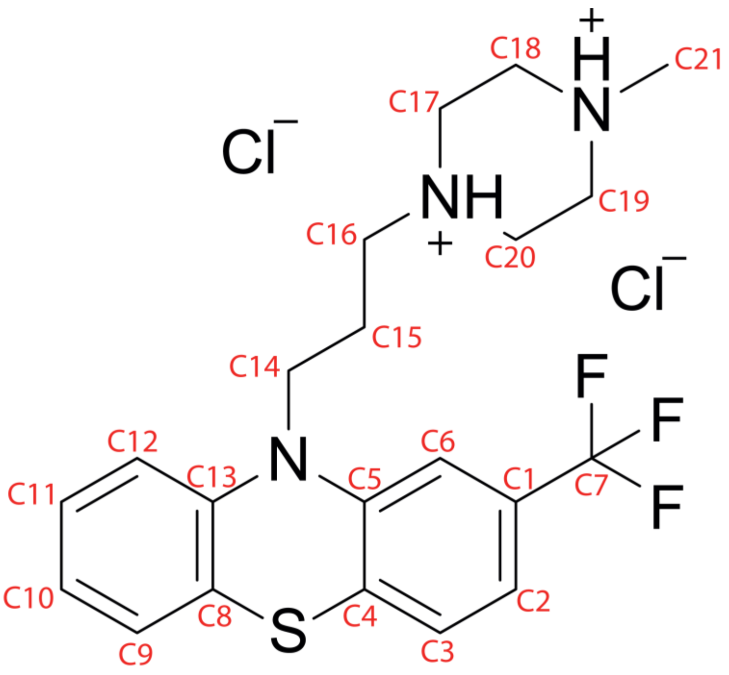

Figure S18. Molecular structure of trifluoperazine dihydrochloride (**3**) showing the carbon atom labels.

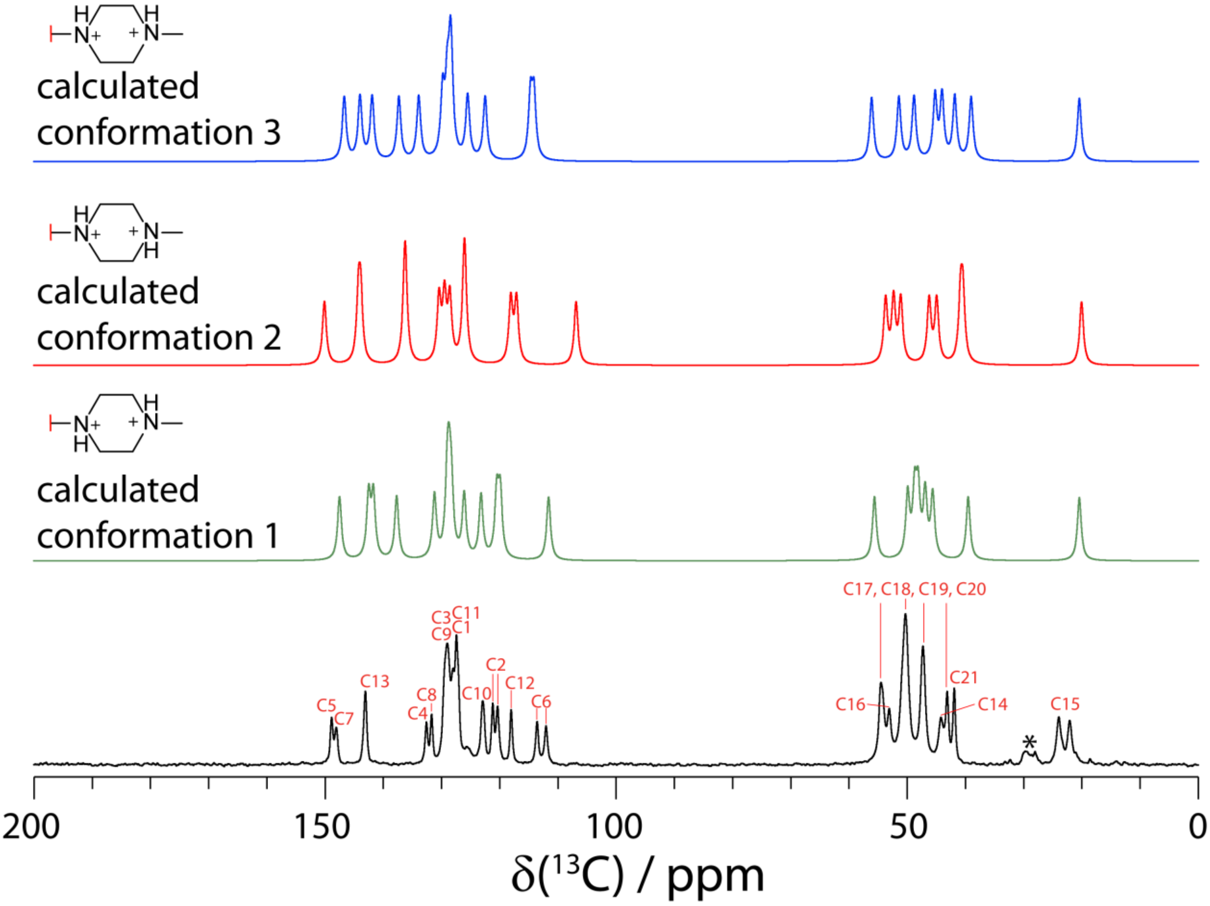

Figure S19. Experimental (black) and GIPAW-calculated (red, green, blue) ^{1}H-^{13}C solid-state NMR CPMAS spectrum of **3** (ν_L = 125.8 MHz, ν_{MAS} = 12.5 kHz). The asterisks denote spinning sidebands, and tentative assignments are given in red. As shown by the molecular structures on the left, the GIPAW calculations were performed on three models of **3** where the piperazinium ring is: puckered down-up (conformation 1), puckered up-down (conformation 2), and puckered up-up (conformation 3). See Figure S15 for more information on the conformations.

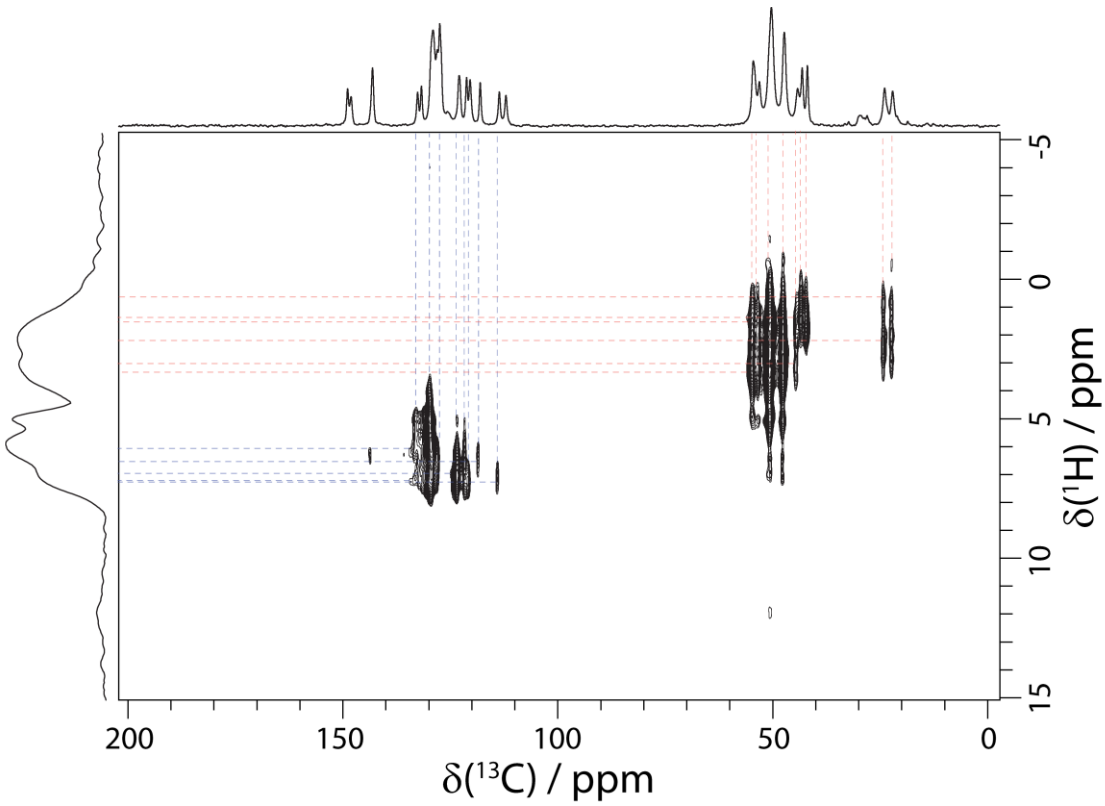

Figure S20. A ^{1}H-^{13}C CP-HETCOR solid-state MAS NMR spectrum with ^{1}H FSLG decoupling of trifluoperazine dihydrochloride (**3**, contact time = 250 µs, ν_L = 500 MHz, ν_{MAS} = 12.5 kHz). The dashed lines are added as a guide. The horizontal axis displays an experimental ^{13}C CPMAS spectrum, while the vertical axis is a skyline projection. 36 transients were collected per t_1 FID, acquiring 192 FIDs in the indirect dimension and using the States-TPPI acquisition mode.

Table S5. Experimental and GIPAW-DFT calculated ^{13}C chemical shifts of **3**. The GIPAW calculations were performed on **3** with atoms in either position 1, 2, or 3 (see Figure S19).[a] The assignments are tentative due to the crystallographic disorder.

atom label	experimental $\delta_{iso}(^{13}C)$ / ppm	calculated $\delta_{iso}(^{13}C)$ position 1 / ppm	calculated $\delta_{iso}(^{13}C)$ position 2 / ppm	calculated $\delta_{iso}(^{13}C)$ position 3 / ppm
C1	127.4	126.3	117.9	128.1
C2	120.3	120.7	125.8	122.0
C3	129.0	128.9	136.0	128.5
C4	132.5	137.9	129.3	136.9
C5	148.8	147.7	149.9	146.3
C6	112.0	111.8	106.7	113.7
C7	148.0	142.7	143.7	143.6
C8	131.7	131.4	136.1	133.5
C9	129.0	129.2	130.2	129.3
C10	122.9	123.4	125.9	125.1
C11	128.0	128.5	128.3	127.8
C12	118.0	120.1	116.9	114.2
C13	143.0	141.9	144.0	141.4
C14	44.2	47.3	41.5	45.4
C15	23.9	20.9	21.1	21.8
C16	53.0	56.0	54.8	52.8
C17	54.5[b]	46.0	46.0	57.5
C18	50.3[b]	49.2	53.4	43.3
C19	47.3[b]	48.5	47.3	46.6
C20	43.1[b]	50.3	52.2	50.2
C21	41.9	39.9	41.9	40.5

[a] σ_{calc} converted to δ_{calc} using $\delta_{calc} = \sigma_{ref} - \sigma_{calc}$ where:[4]

(*position 1*) $\sigma_{ref}(^{13}C)$ = 170.1 ppm for chemical shifts above 100 ppm, $\sigma_{ref}(^{13}C)$ = 171.4 ppm for chemical shifts below 100 ppm

(*position 2*) $\sigma_{ref}(^{13}C)$ = 171.3 ppm for chemical shifts above 100 ppm, $\sigma_{ref}(^{13}C)$ = 171.5 ppm for chemical shifts below 100 ppm

(*position 3*) $\sigma_{ref}(^{13}C)$ = 170.7 ppm for chemical shifts above 100 ppm, $\sigma_{ref}(^{13}C)$ = 172.6 ppm for chemical shifts below 100 ppm

[b] Ambiguous assignment due to the crystallographic disorder.

3.5 – ^{1}H Solid-State NMR

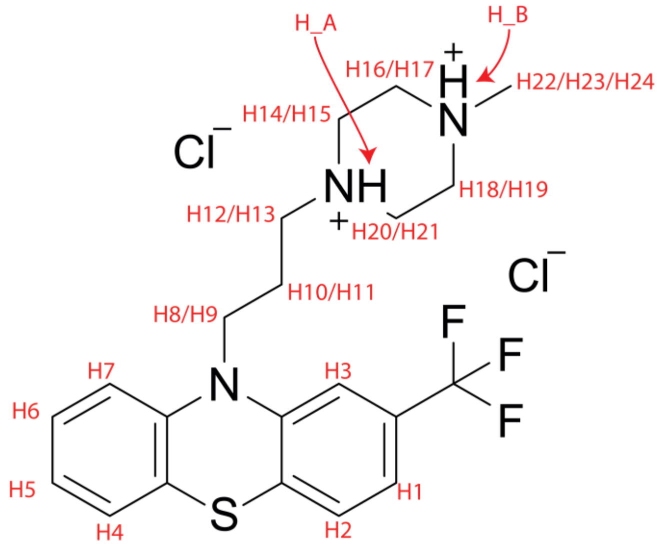

Figure S21. Molecular structure of trifluoperazine dihydrochloride (**3**) showing the hydrogen atom labels. Atoms H_A and H_B were missing atoms added during the process of structural modelling.

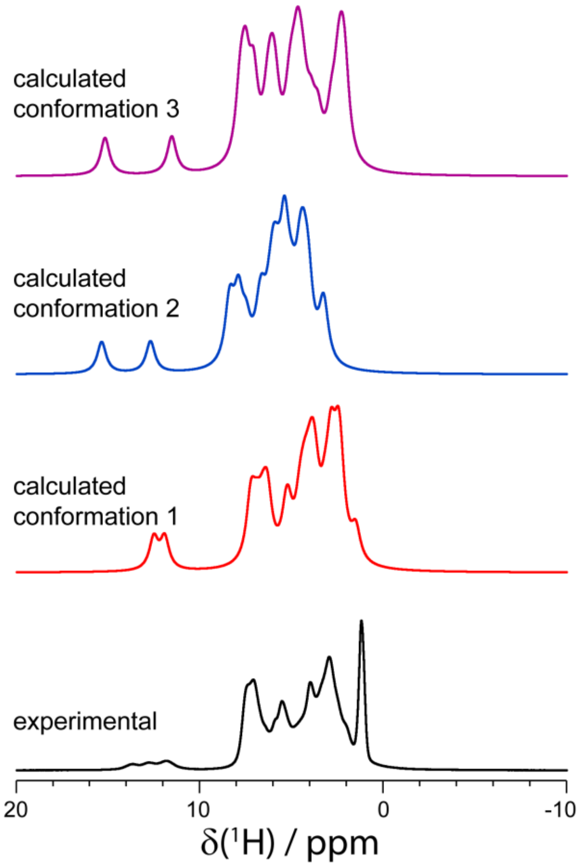

Figure S22. Experimental (black) and GIPAW-calculated (red, blue, purple) ^{1}H solid-state NMR MAS spectrum of **3** at 20.0 T (ν_L = 850.2 MHz, ν_{MAS} = 60 kHz). The GIPAW calculations were performed on three models of **3** where the piperazinium ring is: puckered down-up (conformation 1), puckered up-down (conformation 2), and puckered up-up (conformation 3). See Figure S15 for more information on the conformations.

Table S6. Experimental and GIPAW-DFT calculated ^{1}H chemical shifts of **3**. The GIPAW calculations were performed on **3** with atoms in either position 1, 2 or 3 (see Figure S19).[a] The assignments are tentative due to the crystallographic disorder.

atom label	experimental $\delta_{iso}(^1H)$ / ppm[b]	calculated $\delta_{iso}(^1H)$ position 1 / ppm	calculated $\delta_{iso}(^1H)$ position 2 / ppm	calculated $\delta_{iso}(^1H)$ position 3 / ppm
H1	7.0	7.8	6.0	7.4
H2	7.0	6.0	5.8	6.1
H3	7.0	7.2	6.7	7.8
H4	7.0	6.9	7.6	7.0
H5	7.0	7.9	7.2	7.6
H6	7.0	7.5	7.6	7.4
H7	7.0	7.0	7.1	7.0
H8	3.9	4.3	4.5	4.5
H9	3.9	4.5	3.3	4.6
H10	3.9	3.8	2.4	2.3
H11	3.9	2.2	2.5	2.4
H12	5.5	3.5	4.6	4.3
H13	5.5	3.5	4.7	5.8
H14	2.9	3.2	5.2	2.1
H15	2.9	5.2	4.6	4.7
H16	2.9	4.5	5.4	6.3
H17	2.9	4.7	3.3	2.2
H18	2.9	3.6	5.0	5.1
H19	2.9	4.9	4.2	4.9
H20	2.9	5.8	3.8	5.9
H21	2.9	5.0	5.2	3.9
H22	1.2	3.0	3.7	2.8
H23	1.2	3.0	3.5	3.5
H24	1.2	3.1	3.6	2.0
H_A[c]	11.8, 12.7, 13.6	13.2	11.9	11.5
H_B[c]	11.8, 12.7, 13.6	12.6	14.6	15.1

[a] σ_{calc} converted to δ_{calc} using $\delta_{calc} = \sigma_{ref} - \sigma_{calc}$ where: (*position 1*) $\sigma_{ref}(^1H)$ = 30.2 ppm, (*position 2*) $\sigma_{ref}(^1H)$ = 30.9 ppm, (*position 3*) $\sigma_{ref}(^1H)$ = 30.5 ppm

[b] Ambiguous assignment due to the crystallographic disorder and spectral overlap.

[c] Atoms H_A and H_B were missing atoms added during the process of structural modelling.

3.6 – ^{35}Cl Solid-State NMR

Table S7. GIPAW-DFT calculated ^{35}Cl parameters for **3** in the three distinct conformations.

	**conformation 1**		**conformation 2**		**conformation 3**	
parameter	Cl(1)	Cl(2)	Cl(1)	Cl(3)	Cl(2)	Cl(3)
$\lvert C_Q$ / MHz $\rvert$	9.2	8.1	10.5	8.5	13.7	13.4
η	0.41	0.26	0.59	0.59	0.38	0.16
δ_{calc} / ppm[a]	116	110	72	57	38	159

[a] σ_{calc} converted to δ_{calc} using $\sigma_{ref}(^{35}Cl)$ = 962 ppm and $\delta_{calc} = \frac{\sigma_{ref} - \sigma_{calc}}{1 - \sigma_{ref}}$.

References.

[1] M. Bhadbhade, J. Hook, C. Marjo, A. Rich, Q. Lin, *Acta Crystallogr. Sect. Sect. E: Struct. Rep. Online* **2009**, *65*, o2294.

[2] G. Borodi, M. M. Pop, O. Onija, X. Filip, *Cryst Growth Des* **2012**, *12*, 5846-5851.

[3] J. J. H. McDowell, *Acta Cryst.* **1980**, *B36*, 2178-2181.

[4] A. L. Webber, L. Emsley, R. M. Claramunt, S. P. Brown, *J. Phys. Chem. A* **2010**, *114*, 10435-10442.

[5] F. A. Perras, C. M. Widdifield, D. L. Bryce, *Solid State Nucl. Magn. Reson.* **2012**, *45-46*, 36-44.
